# A conserved interdomain microbial network underpins cadaver decomposition despite environmental variables

**DOI:** 10.1038/s41564-023-01580-y

**Published:** 2024-02-12

**Authors:** Zachary M. Burcham, Aeriel D. Belk, Bridget B. McGivern, Amina Bouslimani, Parsa Ghadermazi, Cameron Martino, Liat Shenhav, Anru R. Zhang, Pixu Shi, Alexandra Emmons, Heather L. Deel, Zhenjiang Zech Xu, Victoria Nieciecki, Qiyun Zhu, Michael Shaffer, Morgan Panitchpakdi, Kelly C. Weldon, Kalen Cantrell, Asa Ben-Hur, Sasha C. Reed, Greg C. Humphry, Gail Ackermann, Daniel McDonald, Siu Hung Joshua Chan, Melissa Connor, Derek Boyd, Jake Smith, Jenna M. S. Watson, Giovanna Vidoli, Dawnie Steadman, Aaron M. Lynne, Sibyl Bucheli, Pieter C. Dorrestein, Kelly C. Wrighton, David O. Carter, Rob Knight, Jessica L. Metcalf

**Affiliations:** 1 Department of Animal Sciences, Colorado State University, Fort Collins, CO USA; 2 Department of Microbiology, University of Tennessee, Knoxville, TN USA; 3 Department of Animal Sciences, Auburn University, Auburn, AL USA; 4 Department of Soil and Crop Sciences, Colorado State University, Fort Collins, CO USA; 5 Collaborative Mass Spectrometry Innovation Center, Skaggs School of Pharmacy and Pharmaceutical Sciences, University of California San Diego, San Diego, CA USA; 6 Department of Chemical and Biological Engineering, Colorado State University, Fort Collins, CO USA; 7 Department of Pediatrics, University of California San Diego, La Jolla, California USA; 8 Center for Studies in Physics and Biology, Rockefeller University, New York, NY USA; 9 Institute for Systems Genetics, New York Grossman School of Medicine, New York University, New York, NY USA; 10 Department of Computer Science, New York University, New York, NY USA; 11 Department of Biostatistics and Bioinformatics, Duke University, Durham, NC USA; 12 Department of Computer Science, Duke University, Durham, NC USA; 13 Graduate Program in Cell and Molecular Biology, Colorado State University, Fort Collins, CO USA; 14 School of Food Science and Technology, Nanchang University, Nanchang, Jiangxi China; 15 School of Life Sciences, Arizona State University, Tempe, AZ USA; 16 Center for Fundamental and Applied Microbiomics, Arizona State University, Tempe, AZ USA; 17 Department of Computer Science and Engineering, University of California San Diego, La Jolla, CA USA; 18 Department of Computer Science, Colorado State University, Fort Collins, CO USA; 19 U.S. Geological Survey, Southwest Biological Science Center, Moab, UT USA; 20 Forensic Investigation Research Station, Colorado Mesa University, Grand Junction, CO USA; 21 Forensic Anthropology Center, Department of Anthropology, University of Tennessee, Knoxville, TN USA; 22 Department of Social, Cultural, and Justice Studies, University of Tennessee at Chattanooga, Chattanooga, TN USA; 23 Mid-America College of Funeral Service, Jeffersonville, IN USA; 24 Department of Biological Sciences, Sam Houston State University, Huntsville, TX USA; 25 Laboratory of Forensic Taphonomy, Forensic Sciences Unit, School of Natural Sciences and Mathematics, Chaminade University of Honolulu, Honolulu, HI USA; 26 Center for Microbiome Innovation, University of California San Diego, La Jolla, CA USA; 27 Department of Bioengineering, University of California San Diego, La Jolla, CA USA; 28 Humans and the Microbiome Program, Canadian Institute for Advanced Research, Toronto, Ontario Canada

**Keywords:** Microbiome, Microbial ecology

## Abstract

Microbial breakdown of organic matter is one of the most important processes on Earth, yet the controls of decomposition are poorly understood. Here we track 36 terrestrial human cadavers in three locations and show that a phylogenetically distinct, interdomain microbial network assembles during decomposition despite selection effects of location, climate and season. We generated a metagenome-assembled genome library from cadaver-associated soils and integrated it with metabolomics data to identify links between taxonomy and function. This universal network of microbial decomposers is characterized by cross-feeding to metabolize labile decomposition products. The key bacterial and fungal decomposers are rare across non-decomposition environments and appear unique to the breakdown of terrestrial decaying flesh, including humans, swine, mice and cattle, with insects as likely important vectors for dispersal. The observed lockstep of microbial interactions further underlies a robust microbial forensic tool with the potential to aid predictions of the time since death.

## Abstract (web summary)

A conserved network of bacteria and fungi underpins cadaver decomposition despite selection effects of location, climate and season.

## Main

Decomposition is one of Earth’s most foundational processes, sustaining life through the recycling of dead biological material^1,2^. This resource conversion is critical for fuelling core ecosystem functions, such as plant productivity and soil respiration. Microbial networks underpin organic matter breakdown^3^, yet their ecology remains in a black box, obscuring our ability to accurately understand and model ecosystem function, resilience and biogeochemical carbon and nutrient budgets. While DNA-based assessments of decomposer microbial communities have occurred in plant litter^4,5^ and a few in mammals^6,7^, little has been revealed about the microbial ecology of how decomposer microbial communities assemble, interact or function in the ecosystem. Our understanding of how animal remains, or carrion, decompose is in its infancy due to the historical focus on plant litter, which dominates decomposing biomass globally. Nevertheless, an estimated 2 billion metric tons of high-nutrient animal biomass^8^ contribute substantially to ecosystem productivity, soil fertility, and a host of other ecosystem functions and attributes^9,10^. Carbon and nutrients from carrion biomass can be consumed by invertebrate and vertebrate scavengers, enter the atmosphere as gas, or be metabolized by microbes in situ or via leachate in the surrounding soils^11,12^. The proportion of carrion carbon and nutrients entering each resource pool is not well quantified and probably highly variable with substantial contributions to each at an ecosystem scale^2,13^. Unlike with plant litter, which is primarily composed of cellulose, animal decomposers must predominantly break down proteins and lipids, which require a vastly different metabolic repertoire. How microbial decomposers assemble to break down these organic compounds is not well understood. For plant litter, it has been proposed that functional redundancy allows different communities of microbes to assemble in any given location^14^ and perform similar functions. Alternatively, similar microbial community members, or microbial networks, may assemble across sites to outcompete other community members and thrive on nutrients^15^.

Recent research has demonstrated that microbial community response over the course of terrestrial human cadaver decomposition and across a range of mammals, results in a substantial microbial community change through time that is repeatable across individuals^6,7,16–18^ and appears somewhat similar across different soil types^6^ and robust to scavenger activity^16^. These data suggest the potential for universal microbial decomposer networks that assemble in response to mammalian remains. However, it remains unclear how the effects of environmental variability, such as differences in climate, geographic location and season, may affect the assembly processes and interactions of microbial decomposers. Yet understanding and predicting this assembly is important for our understanding of ecosystems and informs practical applications. For example, profiling microbial succession patterns associated with human remains may lead to a novel tool for predicting the postmortem interval (PMI), which has critical societal impact as evidence for death investigations. Within laboratory experiments^6,18^, as well as field experiments in single locations^6,19^, microbial decomposer community succession is closely linked to PMI at accuracies relevant for forensic applications^6,17,18^, but these studies do not inform questions of microbial variation across sites, climates and seasons. Consequently, a robust understanding of how microbial ecological patterns of mammalian, and specifically human, decomposition vary is critical for using and improving these important forensic tools. Unlocking the microbial ecology black box for mammal decomposition, or more generally carrion decomposition, could provide actionable knowledge for innovation in agriculture and the human death care industry (for example, composting of bodies)^20^, sustainability (for example, animal mass mortality events)^21^ and the forensic sciences (for example, estimating PMI)^22^, as well as guide future research on plant decomposition and maintaining global productivity under anthropogenic change.

**Fig. 1:**
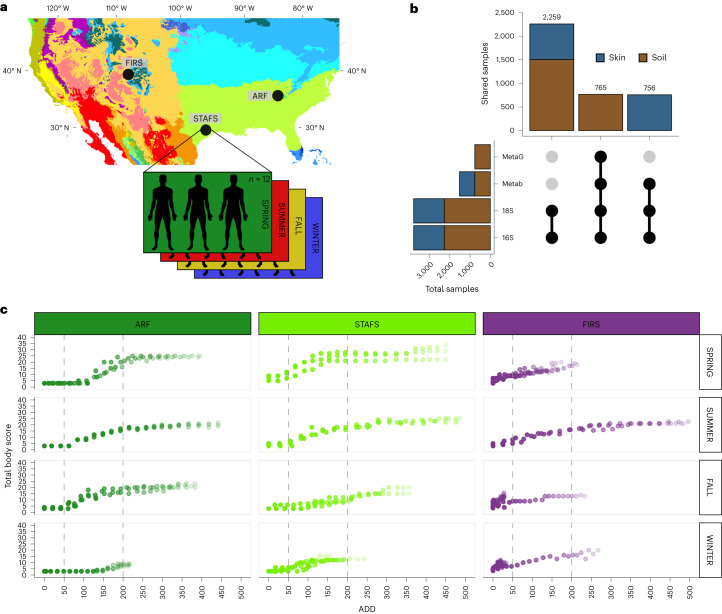
Summary of study design. **a**, Köppen–Geiger climate map showing ARF and STAFS as ‘temperate without a dry season and hot summer’ and FIRS as ‘arid steppe cold’ adapted from ref. ^23^. Thirty-six cadavers in total were placed (*N* = 36), 3 per season for a sum of 12 at each location. **b**, Upset plot representing the experimental design for the total sample size (*x* axis) and number of shared/paired samples (*y* axis) for each data type. MetaG, metagenomics; Metab, metabolomics; 18S, 18S rRNA amplicon; 16S, 16S rRNA amplicon. **c**, Total body score, a visual score of decomposition calculated over the course of decomposition^27^, illustrating how decomposition progresses at each location and by season in triplicate. Dashed lines separate sections of early, active and advanced stages of decomposition as determined by a temperature-based unit of time, accumulated degree day (ADD), calculated by continuously summing the mean daily temperature above 0 °C from left to right. Point transparency increases with days since placement. Source data

**Extended Data Fig. 1:**
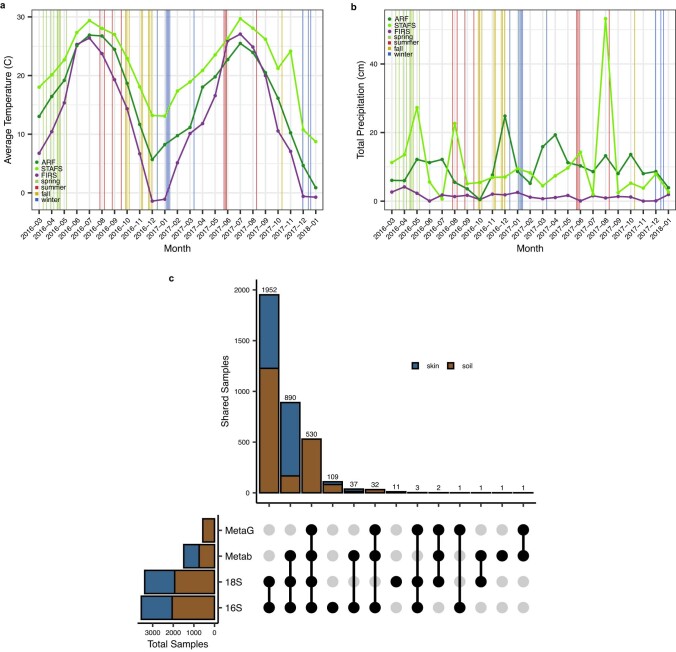
Study Information. Average **a**) temperature data and **b**) total precipitation per location over experiment with cadaver placement dates. Temperature data was collected from local weather stations reported to the National Centers for Environmental Information. Total monthly precipitation data was collected from Weather Underground. The vertical line represents the date of placement and line color denotes the season the body placement is considered to have been placed. **c**) Upset plot illustrating the intersections between sample and omic types after extractions, processing and quality filtering that were used for further analyses. MetaG = metagenomics, Metab = metabolomics, 18S = 18S rRNA amplicon, and 16S = 16S rRNA amplicon. Source data

To address ecological and forensic research questions on decomposer network assembly and function, we used three willed-body donation anthropological facilities in terrestrial environments across two climate types within the United States (Fig. 1a and Extended Data Fig. 1a,b)^23^. We asked whether temporal trends in microbial decomposer communities that we previously characterized in a limited experiment using human cadavers at a single geographic location^6^ were generalizable across climate, geographic locations and seasons. Over the course of decomposition, we compared the microbial response to decomposition across 36 human bodies within (temperate forest) and between (temperate forest vs semi-arid steppe) climate types. We used multi-omic data (16S and 18S ribosomal (r)RNA gene amplicons, metagenomics and metabolomics) to reveal microbial ecological responses to cadaver decomposition over the first 21 d postmortem (Fig. 1b and Extended Data Fig. 1c), when decomposition rates are generally fast and dynamic (Fig. 1c, metadata in Supplementary Table 1). Here we show that a universal microbial decomposer network assembles despite location, climate and seasonal effects, with evidence of increased metabolic efficiencies to process the ephemeral and abundant lipid- and protein-rich compounds. Key members of the microbial decomposer network are also found associated with swine, cattle and mouse carrion^16,24–26^, suggesting that they are not human-specific, but probably general to mammal or animal carrion. Furthermore, the universal microbial network communities underlie a robust microbial-based model for predicting PMI.

## Results

### Nutrient-rich cadaver decomposition

**Extended Data Fig. 2:**
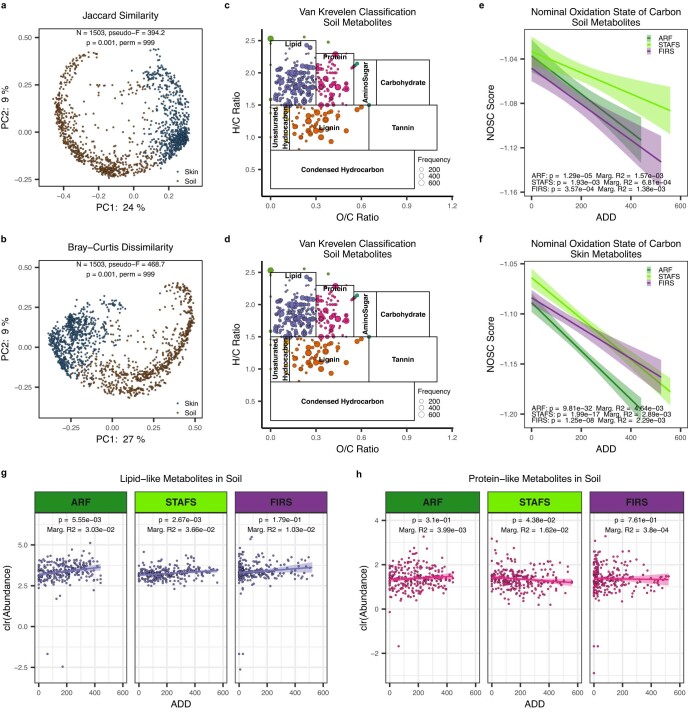
Metabolome Comparison. Principal coordinate analysis (PCoA) of **a**) Jaccard and **b**) Bray-Curtis distances of all unique metabolites and all metabolomic samples show cadaver skin and cadaver-associated soil are significantly different community profiles. n = 1503 biologically independent samples. Significance was determined by PERMANOVA (permutations = 999). Van Krevelen diagram showed a strong presence of lipid-like, protein-like, and lignin-like classes within **c**) cadaver-associated soils and **d**) cadaver skin. Metabolites that matched database chemical formulas or had a significantly predicted chemical formula were assigned a Van Krevelen organic compound classification by their hydrogen:carbon and oxygen:carbon molar ratios. Colors correspond to organic compound classification. Nominal oxidation state of carbon (NOSC) scores for cadaver-associated **e**) soil and **f**) cadaver skin metabolites with assigned chemical formulas show significant decrease of thermodynamic favorability at all geographical locations over decomposition time measured by accumulated degree days (ADD). Soil: ARF n = 251, STAFS n = 250, and FIRS n = 245 biologically independent samples. Skin: ARF n = 250, STAFS n = 249, and FIRS n = 249 biologically independent samples. Data are presented as mean values +/− 95% CI. Significance measured with linear mixed-effects models within each location and adding a random intercept for cadavers with two-tailed ANOVA and no multiple comparison adjustments. **g**) Lipid-like metabolites show an increased abundance in cadaver-associated soils over decomposition measured by accumulated degree days (ADD) and significantly increase in temperate soils. **h**) Protein-like metabolites are less abundant than lipid-like metabolites in cadaver-associated soils over decomposition measured by accumulated degree days (ADD) and significantly decrease in STAFS soil. ARF n = 251, STAFS n = 250, and FIRS n = 245 biologically independent samples. Data are presented as mean values +/− 95% CI. Significance measured with linear mixed-effects models within each location and adding a random intercept for cadavers with two-tailed ANOVA and no multiple comparison adjustments. Metabolite abundance normalized by center log ratio transformation. Source data

Terrestrial mammalian decomposition is a dynamic process that is partly governed by environmental conditions^1,2^. We observed that cadavers placed in the same climate (temperate) decomposed similarly across locations within a season, as determined by a visual total body score (TBS) of decomposition progression (Fig. 1c)^27^. Cadavers placed in a semi-arid climate (that is, FIRS) generally progressed more slowly through decomposition over the 21 d, which is probably due to decreased temperatures, humidity and precipitation in the semi-arid environment (Extended Data Fig. 1a,b)^9,28^. We observed visual cadaver decomposition progression to be impacted by season, wherein summer was the most consistent across locations (Fig. 1c). As cadavers and mammalian carrion decompose, they release a complex nutrient pool that impacts the surrounding environment, often resulting in the death and restructuring of nearby plant life^2,29^ due to generally high inputs of nitrogen^2,6,9,30,31^, which is primarily in the form of ammonium^6^, as well as carbon^2,6,10,30,31^ and phosphorous^9,29^. We characterized the cadaver-derived nutrient pool via untargeted metabolomics using liquid chromatography with tandem mass spectrometry (LC–MS/MS) data. Cadaver skin and associated soil metabolite profiles were distinct (Extended Data Fig. 2a,b). Overall, profiles were largely dominated by likely cadaver-derived lipid-like and protein-like compounds, along with plant-derived lignin-like compounds (Extended Data Fig. 2c,d). As decomposition progressed, both cadaver-associated soil and skin profiles became enriched in linoleic acids, aleuritic acids, palmitic acids, long-chain fatty acids, fatty amides and general amino acids (Supplementary Tables 2 and 3). Furthermore, we estimated a reduction of thermodynamic favourability in the nutrient pool at all locations (Extended Data Fig. 2e,f), a similar pattern found in the microbial breakdown of plant material in soils^32^. These data suggest that during the first weeks of decomposition, more recalcitrant lipid-like and lipid-derivative nutrients build up within soils as decomposers preferentially utilize labile protein-like resources, but with climate-dependent abundance variations in lipid-like (Extended Data Fig. 2g) and geographic-dependent variations in protein-like compounds (Extended Data Fig. 2h). These patterns may also be influenced by the physical properties of soil at each location such as texture, density and stoichiometry.

### Cadaver microbial decomposer assembly

**Fig. 2:**
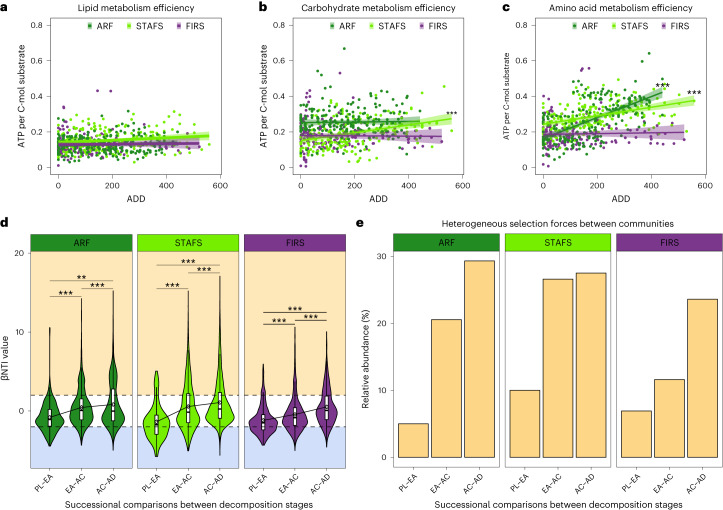
Decomposer community assembly is governed by stochastic and deterministic bacterial assembly processes. **a**–**c**, Lipid (**a**), carbohydrate (**b**) and amino acid (**c**) metabolism efficiency as determined by the maximum ATP per C-mol of substrate that can be obtained from each community, plotted against the ADD the community was sampled. ARF *n* = 212, STAFS *n* = 198 and FIRS *n* = 158 biologically independent samples. Data are presented as mean ± 95% confidence interval (CI). Significance was tested with linear mixed-effects models within each location including a random intercept for cadavers with two-tailed ANOVA and no multiple-comparison adjustments. ARF amino acids *P* = 6.27 × 10^−23^, STAFS amino acids *P* = 6.626 × 10^−10^, STAFS carbohydrate *P* = 2.294 × 10^−07^ and STAFS lipid *P* = 3.591 × 10^−02^. **d**, Pairwise comparisons to obtain βNTI values focused on successional assembly trends by comparing initial soil at time of cadaver placement to early decomposition soil, then early to active and so on (PL, placement; EA, early; AC, active; AD, advanced) in the 16S rRNA amplicon dataset, showing that strong selection forces are pushing the community to differentiate. ARF *n* = 232, STAFS *n* = 202 and FIRS *n* = 182 biologically independent samples. In boxplots, the lower and upper hinges of the box correspond to the first and third quartiles (the 25th and 75th percentiles); the upper and lower whiskers extend from the hinge to the largest and smallest values no further than 1.5× interquartile range (IQR), respectively; and the centre lines represent the median. The βNTI mean (diamond symbol) change between decomposition stage is represented by connected lines. Dashed lines represent when |βNTI| = 2. A |βNTI| value < 2 indicates stochastic forces (white background) drive community assembly. βNTI values <−2 and >2 indicate homogeneous (blue background) and heterogeneous (yellow background) selection drive assembly, respectively. The width of the violin plot represents the density of the data at different values. Significance was tested with Dunn Kruskal–Wallis *H*-test, with multiple-comparison *P* values adjusted using the Benjamini–Hochberg method. **e**, Representation of heterogeneous selection pressure relative abundance within the total pool of assembly processes increases over decomposition in the 16S rRNA amplicon dataset. Bars were calculated by dividing the number of community comparisons within with βNTI > +2 by the total number of comparisons. *P < 0.05, **P < 0.01 and ***P < 0.001. Source Data

**Fig. 3:**
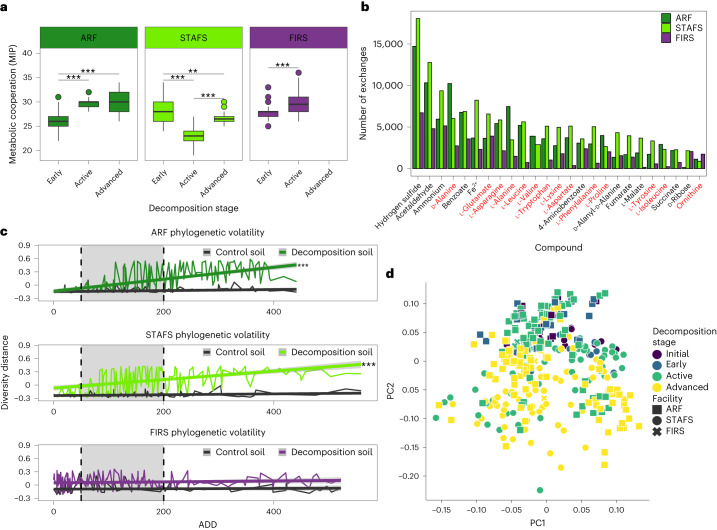
Decomposition microbial ecology is influenced by microbial interactions and environmental conditions. **a**, Predicted cross-feeding interactions from MAGs are site-specific and significantly altered over decomposition. ARF *n* = 201, STAFS *n* = 188 and FIRS *n* = 151 biologically independent samples. In boxplots, the lower and upper hinges correspond to the first and third quartiles (the 25th and 75th percentiles); the upper and lower whiskers extend from the hinge to the largest and smallest values no further than 1.5× IQR; the centre lines represent the median. Significance was tested with Dunn Kruskal–Wallis *H*-test, with multiple-comparison *P* values adjusted with the Benjamini–Hochberg method. ARF early-active *P* = 1.95 × 10^−23^, early-advanced *P* = 1.67 × 10^−23^; STAFS early-active *P* = 5.53 × 10^−39^, early-advanced *P* = 3.65 × 10^−03^, active-advanced *P* = 2.04 × 10^−24^; FIRS early-active *P* = 3.81 × 10^−15^. **b**, Increased cross-feeding reactions during semi-arid active decomposition and temperate advanced decomposition are summarized to show that compounds such as amino acids (red) are common among the top 25 potential cross-fed molecules from MAGs. **c**, Phylogenetic turnover in decomposition soil vs control soil shows that temperate climates react quickly to decomposition, while the more arid site does not quickly change (dashed lines represent breaks for early, active (grey shading) and advanced decomposition stages) using the 16S rRNA gene amplicon dataset. ARF *n* = 414, STAFS *n* = 316 and FIRS *n* = 310 biologically independent samples. Data are presented as mean ± 95% CI. Significance was tested using linear mixed-effects models within each location, including a random intercept for cadavers with two-tailed ANOVA and no multiple-comparison adjustments. ARF and STAFS richness *P* ≤ 2 × 10^−16^. **d**, Multi-omic (16S rRNA gene abundances, 18S rRNA gene abundances, MAG abundances, MAG gene abundances, MAG gene functional modules and metabolites) joint-RPCA shows that microbial community ecology is impacted by decomposition stage and geographical location. **P < 0.01 and ***P < 0.001. Source data

**Extended Data Fig. 3:**
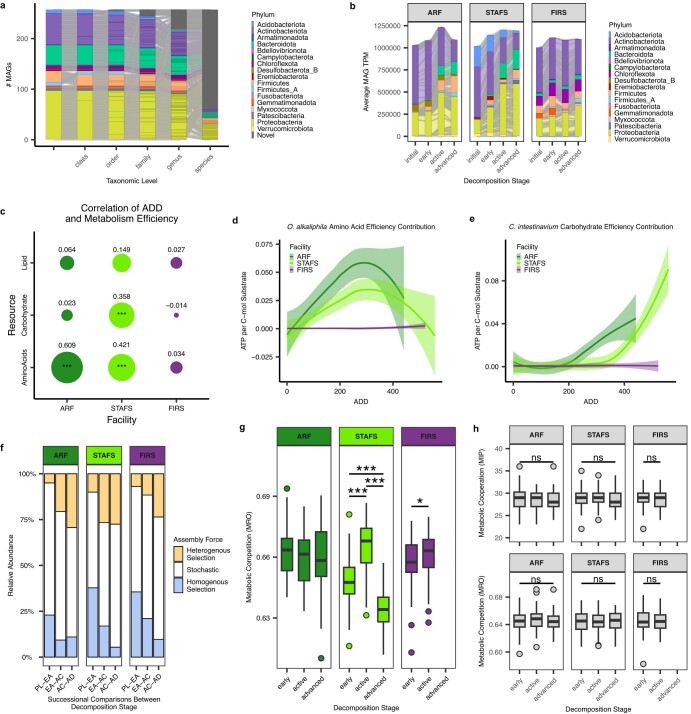
Community Assembly. Sankey diagram of the **a**) 257 99% dereplicated, medium to high quality MAGs with Genome Taxonomy Database classifications and **b**) the average MAG abundances (given as transcript per million, TPM) at each decomposition stage within each location. Proteobacteria and Bacteroidota representation increases with decomposition while Actinobacteria representation decreases at each location. This MAG set encompassed novel bacterial orders (n=3), families (n=9), genera (n=28), and species (n=158). Proteobacteria is the highest represented phylum. **c**) Spearman correlation of the maximum ATP per C-mol for lipids, carbohydrates, and amino acids over ADD at each location represented by circle size. Metabolism efficiency is correlated with ADD in temperate climates. ARF n = 212, STAFS n = 198, and FIRS n = 158 biologically independent samples. Significance measured with linear mixed-effects models within each location and adding a random intercept for cadavers and denoted as p<0.05 (*), p<0.01 (**), and p<0.001 (***). ARF: Amino Acids p = <2e-16, STAFS: Amino Acids p = 1.18e-06, and Carbohydrate p = 4.22e-04. **d**) The amino acid metabolism efficiency of the total community that can be attributed to *O. alkaliphila* and **e**) the carbohydrate metabolism efficiency of the total community that can be attributed to *C. intestinavium* increase over decomposition at temperate locations as a product of the genome’s metabolism efficiency and relative abundance. Data plotted with loess regression as mean values +/− 95% CI. ARF n = 212, STAFS n = 198, and FIRS n = 158 biologically independent samples. **f**) Pairwise comparisons to obtain beta nearest taxon index (βNTI) values focused on successional assembly trends by comparing initial non-decomposition soil to early decomposition soil then early to active, etc. (PL = placement, EA = early, AC = active, AD = advanced) in the 16S rRNA amplicon dataset. Relative abundance of assembly forces reveals that heterogeneous selection (βNTI > +2) pressure increases and homogenous selection (βNTI < -2) decreases over decomposition. Stochastic forces are a constant driver of community assembly (+2 > βNTI > -2). **g**) Predicted metabolic competition from metagenome-assembled genomes are site-specific and significantly altered over decomposition. STAFS: early-active p = 3.42e-11, early-advanced p = 1.23e-11, active-advanced p = 7.85-41, FIRS: early-active p = 0.042. **h**) Predicted metabolic cooperation and competition from metagenome-assembled genomes randomly subsampled into 20-member communities within each site and decomposition serves as a null model comparison signifying the importance of MAG co-occurrence. ARF n = 201, STAFS n = 188, and FIRS n = 151 biologically independent samples. The lower and upper hinges of the boxplot correspond to the first and third quartiles (the 25th and 75th percentiles). The upper whisker extends from the hinge to the largest value no further than 1.5 * IQR from the hinge, and the lower whisker extends from the hinge to the smallest value at most 1.5 * IQR of the hinge. The center of the boxplot is represented by the median. Significance measured with Dunn Kruskal-Wallis H-test with multiple comparison p-values adjusted with the Benjamini-Hochberg method as denoted by p<0.05 (*), p<0.01 (**), and p<0.001 (***). Source data

**Extended Data Fig. 4:**
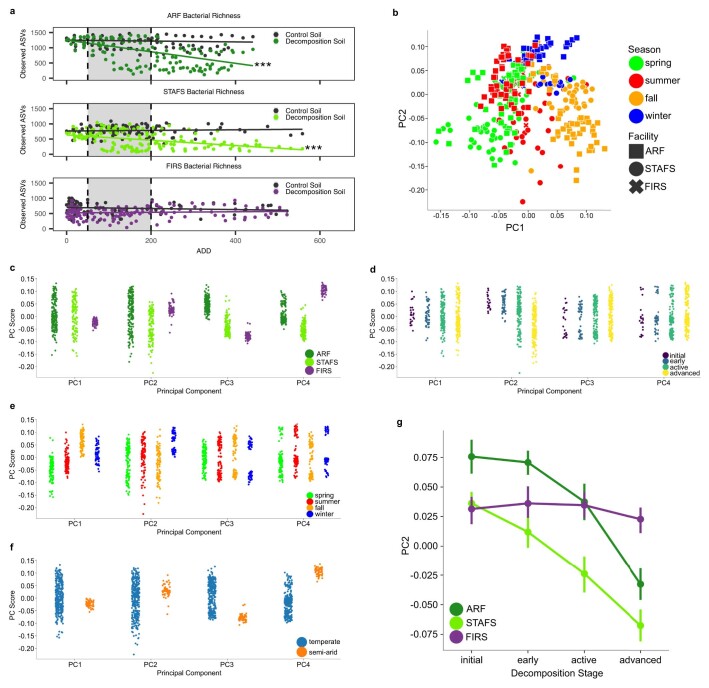
Multi-omic Integration. **a**) ASV richness comparison between decomposition soil and control soil over the decomposition time frame reveals that bacterial richness decreases significantly at temperate locations. ARF n = 414, STAFS n = 316, and FIRS n = 310 biologically independent samples. Significance measured with linear mixed-effects models within each location and adding a random intercept for cadavers with two-tailed ANOVA and no multiple comparison adjustments. ARF and STAFS richness p = <2e-16. Denoted as p<0.05 (*), p<0.01 (**), and p<0.001 (***). **b**) Multi-omic joint-RPCA shows that microbial community ecology is impacted by season and geographical location. Multi-omic Joint-RPCA incorporates soil 16S rRNA, 18S rRNA, metabolomic, and metagenome-assembled genome data. All data types used the same n = 374 biologically independent samples. Multi-omics joint-RPCA principal component scores show that **c**) facility variation is primarily explained by principal component 3 (PC3) and PC4, **d**) decomposition stage is primarily explained by PC2, **e**) season is primarily explained by PC1, and **f**) climate is primarily explained by PC3 and PC4 as described by the least overlap of PC values between groups. **g**) PC2 from the multi-omics joint-RPCA scores for each geographical location over decomposition stages shows the temperate climate locations are the most dynamic in their microbial ecology. Multi-omic Joint-RPCA incorporates soil 16S rRNA, 18S rRNA, metabolomic, and metagenome-assembled genome data. All data types used the same n = 374 biologically independent samples. Data in panel g are presented as mean values +/− 95% CI. Source data

The lipid- and protein-rich cadaver nutrient influx is a major ecological disturbance event that attracts scavengers from across the tree of life and initiates the assembly of a specific microbial decomposer community. On the basis of our metabolite data, we hypothesized that soil decomposer microbial communities preferentially shift to efficiently utilize more labile compounds (for example, amino acids from proteins and possibly also carbohydrates such as glycogen, which were not detected via LC–MS/MS metabolomics) and temporarily leave the less-labile compounds (for example, lipids) in the system. By building a metagenome-assembled genome (MAG) database from human decomposition-associated soils (Extended Data Fig. 3a,b and Supplementary Tables 4–6), we reconstructed genome-scale metabolic models to characterize how potential metabolic efficiencies of soil microbial communities shift in response to three major resources: lipids, amino acids and carbohydrates. Indeed, we found that temperate decomposer metabolic efficiency of labile resources was positively correlated with a temperature-based timeline of decomposition (accumulated degree day (ADD)) (Fig. 2a–c, Extended Data Fig. 3c and Supplementary Tables 7–9). We found that two MAGs constituted a large portion of the increased amino acid and carbohydrate metabolism efficiencies at temperate locations: *Oblitimonas alkaliphila* (*Thiopseudomonas alkaliphila*) (Extended Data Fig. 3d) and *Corynebacterium intestinavium* (Extended Data Fig. 3e), respectively. This microbial response is probably an effect of heterogeneous selection (that is, selection driving the community to become different) driving the assemblage of the decomposer community, as heterogeneous selection increases relative to stochastic forces and homogeneous selection during decomposition (Fig. 2d,e, Extended Data Fig. 3f, and Supplementary Tables 10 and 11). We further hypothesized that microbe–microbe interactions probably contribute to selection^33^, which we investigated by calculating metabolic competitive and cooperative interaction potentials between our genome-scale metabolic models^34,35^. We found that metabolic competition potential initially increased at one temperate and the semi-arid location, suggesting an increase in microbes with similar resource needs (Extended Data Fig. 3g, and Supplementary Tables 12 and 13), which was not seen when communities were randomly subsampled within each site and decomposition stage (Extended Data Fig. 3h and Supplementary Table 12). Furthermore, we found that communities in temperate climates increased cross-feeding potential (that is, sharing of metabolic products) from early/active to advanced decomposition (Fig. 3a, and Supplementary Tables 12 and 13) and had a substantially higher number of cross-feeding exchanges during late decomposition than semi-arid climate communities (Fig. 3b and Supplementary Table 14), suggesting the increased potential for metabolic activity. The molecules predicted most for exchange by the models are common by-products of mammalian decomposition^36,37^, specifically of protein degradation^38^, and included hydrogen sulfide, acetaldehyde and ammonium, and 56% of the top 25 total exchanged molecules were amino acids. In contrast to temperate locations, semi-arid decomposer communities demonstrated a relatively diminished responsiveness to decomposition stage (Fig. 3c, Extended Data Fig. 4a, and Supplementary Tables 15 and 16) and did not significantly shift their metabolism efficiencies (Fig. 2a–c, Extended Data Fig. 3c and Supplementary Tables 7–9), probably due to a lack of water, which leads to higher metabolic costs^39^, decreased substrate supply^40^ and growth^41^. Despite a less measurable microbial response at the semi-arid location, we did detect an increase in cross-feeding potential from early to active decomposition stages, suggesting that the semi-arid community has an increased ability to respond to decomposition nutrients (Fig. 3a, and Supplementary Tables 12 and 13) but probably at a smaller scale than temperate locations.

We further investigated potential effects of selective environmental conditions via multi-omic, joint robust principal components analysis (joint-RPCA) for dimensionality reduction (see Methods)^42^, which all (climate, geographic location, season and decomposition stage) significantly shaped the microbial decomposer community ecology (Fig. 3d, Extended Data Fig. 4b–f and Supplementary Table 17). Climate (temperate vs semi-arid) along with location (ARF, STAFS, FIRS) significantly shaped the soil microbial community composition (Supplementary Tables 18–20) and its potential gene function (Supplementary Tables 21–22). Decomposition soils at temperate sites exhibited strong microbial community phylogenetic turnover (Fig. 3c and Supplementary Table 15) and a decrease in microbial richness during decomposition (Extended Data Fig. 4a and Supplementary Table 16), while less measurable effects were observed at the semi-arid location (Fig. 3c, Extended Data Fig. 4a, and Supplementary Tables 15 and 16). Season appeared to primarily influence soil chemistry as opposed to microbial community composition during decomposition (Supplementary Table 23), suggesting possible temperature-associated metabolism changes/limitations of microbial decomposer taxa. Taken together, these data suggest that while stochastic forces play a part in decomposer community assembly, deterministic forces, such as microbial interactions and environmental conditions, also play an important role.

### Conserved interdomain soil microbial decomposer network

**Fig. 4:**
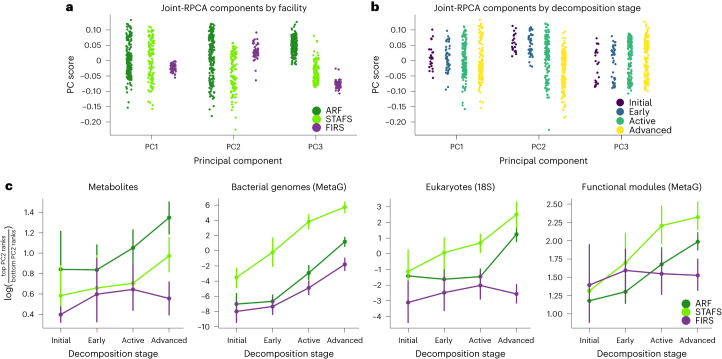
Multi-omics joint-RPCA principal component values. **a**,**b**, Principal component values show that (**a**) facility variation is primarily explained by principal component 3 (PC3) (that is, least overlap between group scores), while variation caused by (**b**) decomposition stage is explained by PC2. **c**, Change in log ratio of PC scores within omics datasets (metabolites, MAG abundances, 18S rRNA gene abundances and MAG gene functional modules) from initial soil through advanced decomposition stage soil highlights that decomposition stage progression corresponds to compositional shifts. All data types used the same *n* = 374 biologically independent samples. Data are presented as mean ± 95% CI. Source data

**Fig. 5:**
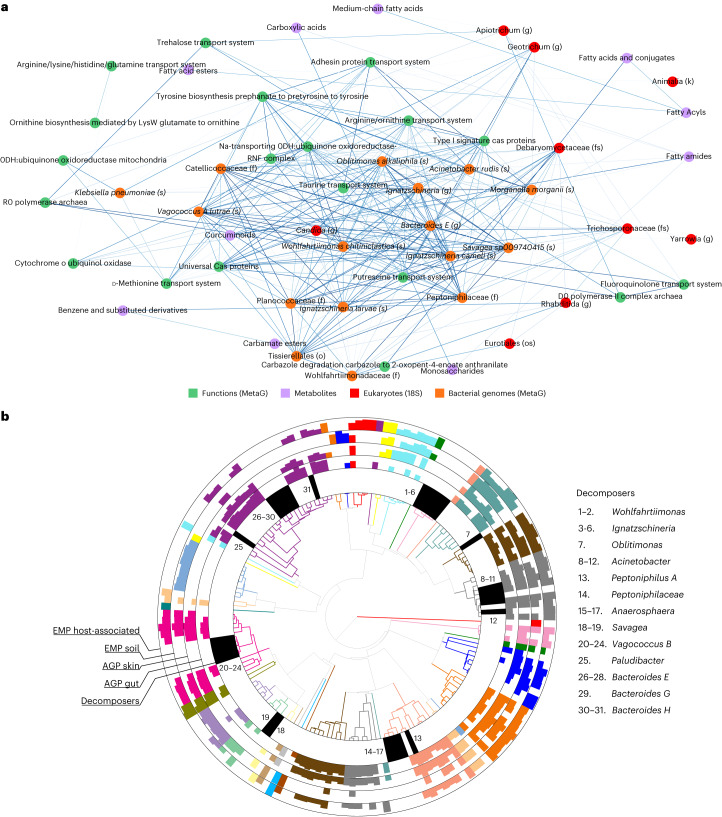
A universal decomposer network emerges across season, location and climate. **a**, Top 20% correlation values from features responsible for the universal late decomposition log-ratio signal in joint-RPCA PC2 visualized in a co-occurrence network. **b**, Phylogenetic tree representing ASVs associated with key decomposer nodes from the network placed along the top 50 most abundant ASVs taken from AGP gut, AGP skin, EMP soil and EMP host-associated datasets demonstrates that key decomposers are largely phylogenetically unique. Colour represents taxonomic order (full legend in Extended Data Fig. 6); the innermost ring represents decomposer placement, while outer rings represent AGP and EMP ASVs, for which bar height represents ASV rank abundance within each environment. A lack of bars indicates that the ASV was not present within the entire dataset. AGP and EMP ASVs were ranked according to the number of samples they were found in each environment. Decomposer ASVs are numbered clockwise (full taxonomy available in Supplementary Table 27). Source Data

**Extended Data Fig. 5:**
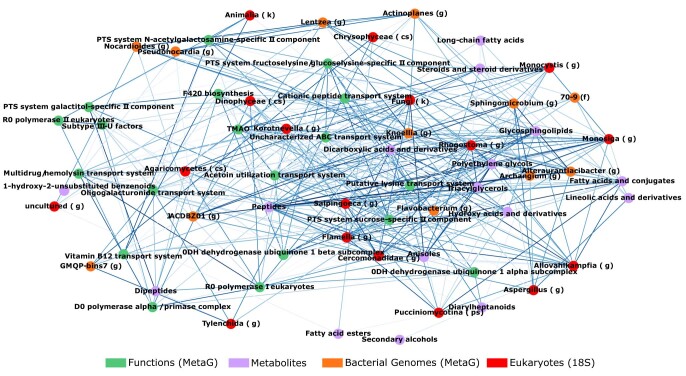
Universal Initial Non-Decomposition And Early Decomposition Soil Network. Top 20% of correlations between selected nodes for the universal initial non-decomposition and early decomposition soil log-ratio signal in Joint-RPCA PC2 visualized in co-occurrences network. All data types used the same n = 374 biologically independent samples. Source data

**Extended Data Fig. 6:**
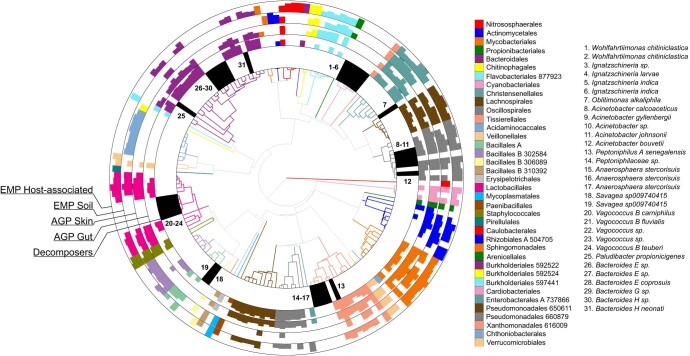
Decomposer ASVs Placed in Current Databases. Phylogenetic tree representing ASVs associated with the key decomposer nodes from the network placed along within the top 50 most abundant ASVs taken from AGP gut, AGP skin, EMP soil, and EMP host-associated datasets demonstrates key decomposers are largely phylogenetically unique. Innermost ring represents decomposer placement while outer rings represent AGP and EMP ASVs, for which bar height represents ASV rank prevalence within each environment. AGP and EMP ASVs were ranked according to the number of samples they were found in each environment. A lack of bars represents that the ASV was not present within the dataset. Decomposer ASVs are numbered clockwise with full taxonomy available in Supplementary Table 27. Source data

We discovered a universal network of microbes responding to the cadaver decomposition despite selection effects of climate, location and season on the assembly of the microbial decomposers within the soil. To focus on the universal decomposition effects across locations, we used the joint-RPCA principal component 2 (PC2) scores to generate the universal decomposition network due to their significant change over decomposition stage and reduced impact from location, season and climate (Fig. 4a,b, Extended Data Fig. 4b–f and Supplementary Table 24). Therefore, PC2 scores were used to calculate multi-omics of log ratios in late decomposition soil compared to initial and early decomposition soils (Fig. 4c, Extended Data Fig. 4g and Supplementary Table 25), which allowed us to identify key co-occurring bacterial and eukaryotic microbial decomposers, bacterial functional pathways and metabolites associated with late decomposition (Fig. 5a, Extended Data Fig. 5 and Supplementary Table 26). The organism *O. alkaliphila*, which is central to the network and a large contributor to the increased amino acid metabolism efficiency at temperate locations (Extended Data Fig. 3d), may play a key role in terrestrial cadaver decomposition as a controller of labile resource utilization in temperate climates, but little is known about its ecology^43–45^. In addition, most microbial key network decomposers (Fig. 5a; *O. alkaliphila*, *Ignatzschineria*, *Wohlfahrtiimonas*, *Bacteroides*, *Vagococcus lutrae, Savagea*, *Acinetobacter rudis* and *Peptoniphilaceae*) represented unique phylogenetic diversity that was extremely rare or undetected in host-associated or soil microbial communities in American Gut Project (AGP) or Earth Microbiome Project (EMP) data sets (Fig. 5b, Extended Data Fig. 6, and Supplementary Tables 27 and 28). Although the decomposers in the group *Bacteroides* have previously been assumed to derive from a human gut source^46,47^, we find that these are instead probably a specialist group of decomposers distinct from gut-associated *Bacteroides* (Fig. 5b, Extended Data Fig. 6, and Supplementary Tables 27 and 28). The only strong evidence of key network bacterial decomposers emerging from soil and host-associated environments were in the genera *Acinetobacter* and *Peptoniphilus* (Fig. 5b, Extended Data Fig. 6, and Supplementary Tables 27 and 28). We more comprehensively characterized microbial decomposer phylogenetic uniqueness with MAG data, which span previously undescribed bacterial orders, families, genera and species (Extended Data Fig. 3a). Overall, we find that the soil microbial decomposer network is phylogenetically unique and in extremely low relative abundance in the environment until the cadaver nutrient pool becomes available.

We hypothesized that specialist decomposer network taxa probably interact to metabolize the nutrient pool, which we explored via estimated cross-feeding capabilities of co-occurring communities. Highlighting the importance of these key taxa, microbial decomposer network members accounted for almost half (42.8%) of predicted late decomposition nutrient exchanges (Figs. 3b and 5a, and Supplementary Table 29) with Gammaproteobacteria being prominent as both metabolite donors and receivers. For example, *O. alkaliphila* has the capability to cross-feed with *Ignatzschineria*, *Acinetobacter*, *Savagea* and *Vagococcus lutrae*, to which it donates amino acids known to be associated with mammalian decomposition such as aspartate, isoleucine, leucine, tryptophan and valine, along with the lipid metabolism intermediate, *sn*-Glycero-3-phosphoethanolamine^36^ (Supplementary Table 30). As a receiver, *O. alkaliphilia* is predicted to receive essential ferrous ions (Fe^2+^) from *Acinetobacter*, *Savagea* and *Vagoccocus* along with glutamate, proline and lysine from *Ignatzschineria*. Further, putrescine, a foul-smelling compound produced during decomposition by the decarboxylation of ornithine and arginine, and arginine/ornithine transport systems were universal functions within our network (Fig. 5a). Cross-feeding analysis identified multiple potential ornithine and/or arginine exchangers, such as *Ignatzschineria*, *Savagea*, *Wohlfahrtiimonas* and *O. alkaliphilia* (Supplementary Table 31). Putrescine is an interdomain communication molecule probably playing an important role in assembling the universal microbial decomposer network by signalling scavengers such as blow flies^48^, which disperse decomposer microbes, as well as directly signalling other key microbial decomposers, such as fungi^49–51^.

Fungi play an essential role in the breakdown of organic matter; however, their processes and interdomain interactions during cadaver decomposition remain underexplored. Our network analysis identified multiple fungal members that are co-occurring with bacteria, belonging to the Ascomycota phylum (Fig. 5a)—a phylum known for its role in breaking down organic matter^6,44,52,53^. In particular, *Yarrowia* and *Candida* are known for their ability to utilize lipids, proteins and carbohydrates^44,53^, and both have one of their highest correlations with *O. alkaliphila* (Fig. 5a and Supplementary Table 25). The ability of *Yarrowia* and *Candida* to break down lipids and proteins during decomposition may serve as interdomain trophic interactions that allow *O. alkaliphila* to utilize these resources^44^. For example, *Yarrowia* and *Candida* genomes contain biosynthesis capabilities for arginine and ornithine that, if excreted, could be taken up by *O. alkaliphilia*. The complete genome of *O. alkaliphilia* (Genbank accession no. CP012358) contains the enzyme ornithine decarboxylase, which is responsible for converting ornithine to the key compound putrescine^43^.

### Machine learning reveals a predictable microbial decomposer ecology

**Fig. 6:**
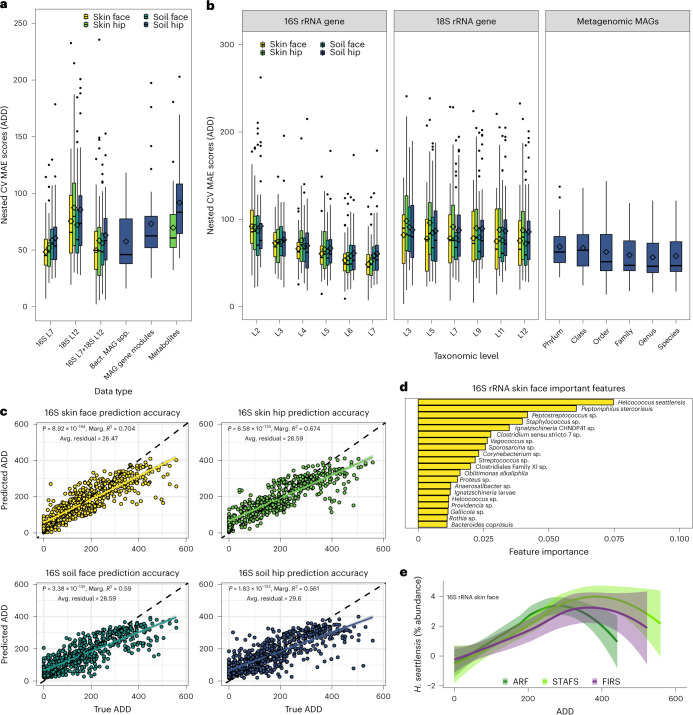
Machine learning reveals predictive nature of microbial communities of time since death (ADD) through universal decomposers. **a**, Cross-validation errors of multi-omic data sets. 16S and 18S rRNA gene data were collapsed to SILVA taxonomic level 7 (L7) and 12 (L12). Boxplots represent average prediction MAE in ADD of individual bodies during nested cross-validation of 36 body dataset. 16S rRNA soil face, soil hip, skin face and skin hip datasets contain *n* = 600, 616, 588 and 500 biologically independent samples, respectively. 18S rRNA soil face, soil hip, skin face and skin hip datasets contain *n* = 939, 944, 837 and 871 biologically independent samples, respectively. Paired 16S rRNA+18S rRNA soil face, soil hip, skin face and skin hip datasets contain *n* = 440, 450, 428 and 356 biologically independent samples, respectively. MAG datasets contain *n* = 569 biologically independent samples. Metabolite soil hip and skin hip datasets contain *n* = 746 and 748 biologically independent samples, respectively. **b**, Mean absolute prediction errors are lowest when high-resolution taxonomic data are used for model training and prediction. Data represented contain the same biologically independent samples as in **a**. In boxplots in **a** and **b**, the lower and upper hinges of the boxplot correspond to the first and third quartiles (the 25th and 75th percentiles); the upper and lower whiskers extend from the hinge to the largest and smallest values no further than 1.5× IQR; the centre lines represent the median; the diamond symbol represents the mean. **c**, Linear regressions of predicted to true ADDs to assess model prediction accuracy show that all sampling locations significantly predict ADD. Data represented contain the same biologically independent samples as in **a**. Data are presented as mean ± 95% CI. Black dashed lines represent ratio of predicted to real ADD predictions at 1:1. The coloured solid lines represent the linear model calculated from the difference between the predicted and real ADD. **d**, The most important SILVA L7 taxa driving model accuracy from the best-performing model derived from 16S rRNA gene amplicon data sampled from the skin of the face. **e**, Comparison of abundance changes of the top important taxon, *Helcococcus seattlensis*, in skin reveals that low-abundance taxa provide predictive responses. Data plotted with loess regression and represent the same biologically independent samples as in **a**. Data are presented as mean ± 95% CI. Bact., bacterial; Avg., average; Marg., marginal. Source data

**Extended Data Fig. 7:**
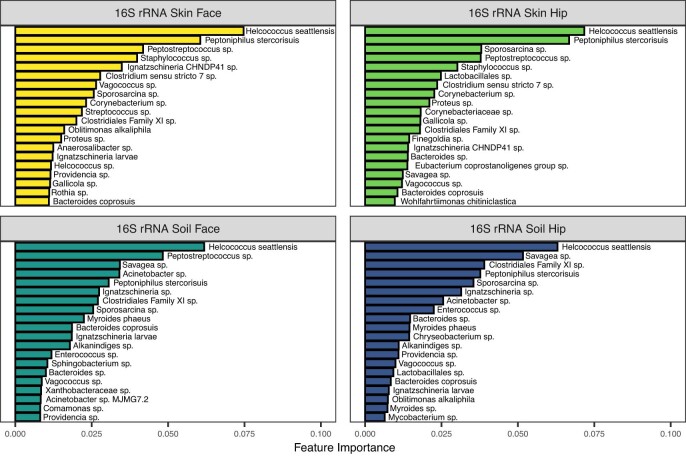
Important Features for 16S rRNA Random Forest Models. The 20 most important SILVA level-7 taxa as determined in the 16S rRNA random forest regression models for predicting postmortem interval shows that many of the same taxa appear important for model prediction within all sample types, but some differences do emerge.

**Extended Data Fig. 8:**
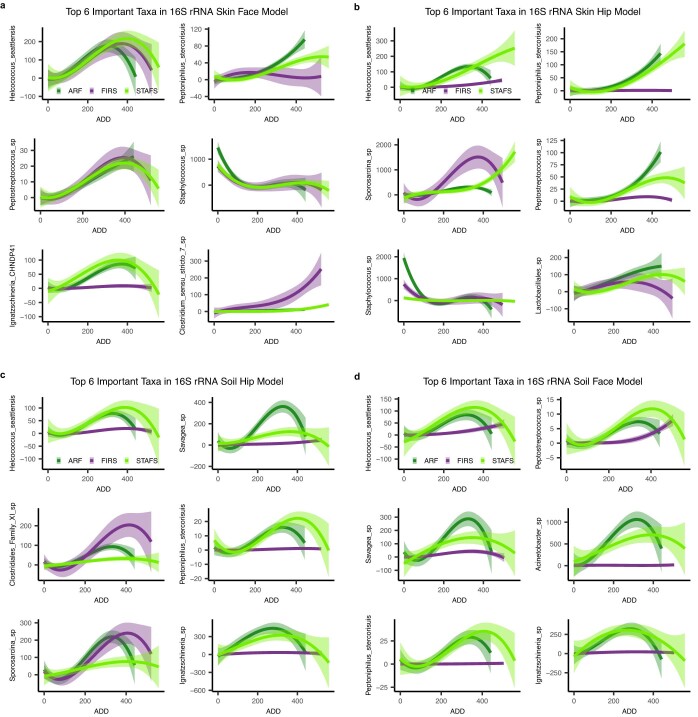
Longitudinal Abundances of Important Features. The 6 most important SILVA level-7 taxa as determined in the 16S rRNA data from the **a**) skin of the face, **b**) skin of the hip, **c**) soil associated with the hip, and **d**) soil associated with the face for random forest regression models for predicting postmortem interval. Data plotted by the taxa and the normalized abundance change over ADD at each geographic location. Data plotted with loess regression and 16S rRNA soil face, soil hip, skin face, and skin hip datasets contain n = 600, 616, 588, and 500 biologically independent samples, respectively. Data are presented as mean values +/− 95% CI.

**Extended Data Fig. 9:**
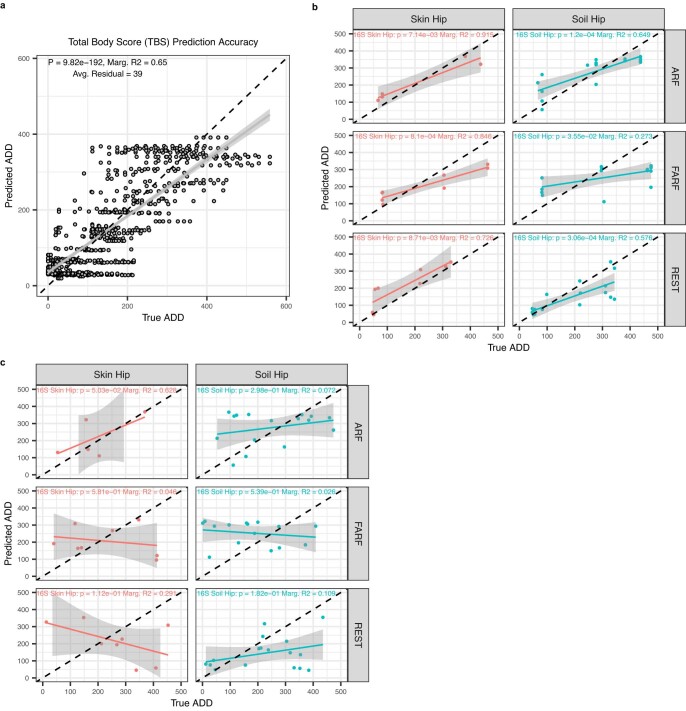
16S rRNA Random Forest Model Validation. **a**) Total body scores (TBS) used to train a random forest model for prediction of PMI (ADD) shows that TBS scores can predict PMI relatively accurately based on a low MAE but have higher variability in their predictions as represented by a higher residual value than microbiome-based models. Models built from 16S rRNA data using SILVA level-7 taxa from the skin and soil associated with the hip were validated with **b**) an independent test set of samples that were collected from cadavers at locations and climates not represented in our model and **c**) the same data where samples were given randomly assigned ADDs within the range of true ADDs to serve as a null model. Significance measured with linear mixed-effects models within each location and adding a random intercept for cadavers with two-tailed ANOVA and no multiple comparison adjustments. Data are presented as mean values +/− 95% CI. Source data

The assembly of a universal microbial decomposer network suggests the potential to build a robust forensics tool. We demonstrate that the PMI (calculated as ADD) can be accurately predicted directly from microbiome-normalized abundance patterns via random forest regression models (Fig. 6a). High-resolution taxonomic community structure was the best predictor of PMI (Fig. 6b), particularly normalized abundances of the 16S rRNA gene at the SILVA database level-7 taxonomic rank (L7) of the skin decomposer microbes (Fig. 6a–c). Interestingly, 3 out of 4 of the skin-associated decomposer taxa that were most informative for the PMI model had similar normalized abundance trends over decompositions for bodies at all locations, suggesting that skin decomposers are more ubiquitous across climates than soil decomposers (Fig. 6d and Extended Data Fig. 7). We hypothesize that this is due to the human skin microbiome being more conserved between individuals than the soil microbiome is between geographic locations^54^. In fact, both skin and soil 16S rRNA-based models had the same top taxon as the most important predictor, *Helcococcus seattlensis* (Fig. 6d and Extended Data Fig. 7). *H. seattlensis* is a member of the order Tissierellales and family Peptoniphilaceae, both of which were key nodes within the universal decomposer network. In line with our hypothesis, *H. seattlensis* on the skin showed more-similar abundance trends for cadavers decomposing across both climate types, while *H. seattlensis* trends in the soil were primarily measurable at temperate locations (Fig. 6e and Extended Data Fig. 8). We found that normalized abundances of important soil taxa previously established to be in our universal decomposer network had strong climate signals, further suggesting a diminished responsiveness in semi-arid climates, such as temperate-climate responses with *H. seattlensis*, *O. alkaliphila*, *Savagea* sp., *Peptoniphilus stercorisuis*, *Ignatzschineria* sp. and *Acinetobacter* sp. (Extended Data Fig. 8c,d). However, we found that the three most important PMI model soil taxa, *Peptostreptococcus* sp., *Sporosarcina* sp. and Clostridiales Family XI sp., had increased detection with decomposition in both semi-arid and temperate climates (Extended Data Fig. 8c,d), suggesting that while strong climate-dependent fluctuations exist, there are microbial members that respond more ubiquitously to decomposition independent of climate. In addition, microbiome-based models and a TBS-based model had comparable average mean absolute errors (MAE) (Extended Data Fig. 9a); however, 16S rRNA microbiome-based model predictions were on average closer to the actual observed values (that is, smaller average residual values), suggesting a higher accuracy (Fig. 6c and Extended Data Fig. 9a). Lastly, we confirmed the model accuracy and reliability of PMI prediction using 16S rRNA amplicon data with an independent test set of samples that were collected at a different time from cadavers at locations and climates not represented in our model. We discovered that we could accurately predict the true PMIs of samples better than samples with randomized PMIs at all independent test set locations (Extended Data Fig. 9b,c and Supplementary Table 32), confirming the generalizability and robustness of our models in predicting new data from multiple geographies and climates with an accuracy useful for forensic death investigations.

## Discussion

We provide a genome-resolved, comprehensive view of microbial dynamics during cadaver decomposition and shed light on the assembly, interactions and metabolic shifts of a universal microbial decomposer network. We found that initial decomposer community assembly is driven by stochasticity, but deterministic forces increase over the course of decomposition, a finding in agreement with other conceptual models of microbial ecology^33,55–57^. These processes led to a decomposer network consisting of phylogenetically unique taxa emerging, regardless of season, location and climate, to synergistically break down organic matter. The ubiquitous decomposer and functional network revealed by our multi-omic data suggests that metabolism is coupled to taxonomy, at least to some extent, for cadaver decomposition ecology. However, the overall composition of microbial decomposer communities did vary between different climates and locations, indicating that some functional redundancy also probably exists. In a study of agricultural crop organic matter decomposition (straw and nutrient amendments), researchers similarly demonstrated that although functional redundancy probably plays a role, key microbial taxa emerge as important plant decomposers^15^, and a meta-analysis of microbial community structure–function relationships in plant litter decay found that community composition had a large effect on mass loss^58^. In terms of climatic controls over cadaver decomposition, temperate locations had a more measurable microbial response (for example, phylogenetic turnover, potential cross-feeding) in soils than the arid location in our study, and plant studies support the idea that climate is a strong determinant of decomposition rates and microbial activity^59^.

Despite the lesser response in the arid location, cadaver decomposer microbial ecologies were similar, suggesting that while climate may act as a strong control, microbial community composition follows similar assembly paths. We find evidence that key interdomain microbial decomposers of cadavers (that is, fungi and bacteria) emerge in diverse environments and probably utilize resource partitioning and cross-feeding to break down a nutrient pulse that is rich in lipids, proteins and carbohydrates. This process would be consistent with dogma within leaf litter ecology that fungal decomposers are typically specialized decomposers of complex substrates while bacteria serve as generalists that decompose a broader nutritional landscape^60^. Thus, we hypothesize that fungi (such as *Yarrowia* and *Candida*) assist in the catabolism of complex, dead organic matter (such as lipids and proteins) into simpler compounds (such as fatty acids and amino acids), which are utilized by bacterial community members, (such as *O. alkaliphila*) capable of efficiently metabolizing these by-products. This division of labour coupled with microbial interactions drives the assembly of the microbial decomposer community, in a process reminiscent of ecological dynamics observed in leaf litter decomposition^60^.

We suspect that key network microbial decomposers are probably not specific to decomposition of human cadavers and are, in part, maintained or seeded by insects. Key cadaver bacterial decomposers *O. alkaliphila*, *Ignatzschineria*, *Wohlfahrtiimonas*, *Bacteroides*, *Vagococcus lutrae*, *Savagea*, *Acinetobacter rudis* and *Peptoniphilaceae* have been detected in terrestrial decomposition studies of swine, cattle and mice (Supplementary Table 33)^16,24–26^, and a subset detected in aquatic decomposition^61^. Most key network bacterial decomposers, including the well-known blow fly-associated genera *Ignatzschineria* and *Wohlfahrtiimonas*^62^, were rare or not detected in a lab-based mouse decomposition study^6^ in which insects were excluded (Supplementary Table 33). However, a different lab-based study that excluded blow flies but included carrion beetles^26^ detected a subset of these key microbial decomposers, suggesting a role for microbe–insect interactions and dispersal by insects^26,48,63^. Further evidence implicating insects as important vectors is that all key network bacterial decomposers presented here have been detected on blow flies (Supplementary Table 28)^6,64^. Furthermore, Ascomycota fungal members, such as *Yarrowia* and *Candida*, have been previously detected in association with human, swine and mouse remains^6,26,44,53^. *Yarrowia* can be vertically transmitted from parent to offspring of carrion beetle^63^ and may facilitate beetle consumption of carrion. Taken together, these findings suggest that key microbial decomposer taxa identified in this study of human cadavers are probably more generalizable carrion decomposers and are likely inoculated, at least partly, by insects.

We demonstrate the potential practical application of microbiome tools in forensic science by leveraging microbial community succession patterns and machine learning techniques for accurately predicting PMI. Importantly, the predictive models showcase their generalizability by accurately predicting the PMIs of independent test samples collected from various geographic locations and climates, including for test samples collected from a climate region not represented in the training set of the model. The best-performing model was able to accurately predict PMI within ~±3 calendar days during internal validation and on an independent test set (Supplementary Tables 34 and 35), which is a useful timeframe for forensic sciences, enabling investigators to establish crucial timelines and aiding in criminal investigations. Prediction errors are probably due to intrinsic (for example, BMI/total mass)^19,24,65^ and/or extrinsic (for example, scavengers, precipitation)^19,26^ factors not accounted for in the model, but should be a future area of research for model improvement. For example, total mass has been previously shown not to affect microbial decomposer composition in swine^24^; however, ref. ^19^ found that Gammaproteobacteria relative abundance correlated with BMI of humans. Within our study, in which cadavers had highly variable initial total masses (Supplementary Table 1), *Acinetobacter* and *Ignatzschineria* (within Gammaproteobacteria) were important features in our PMI models, suggesting that it is probably robust to BMI (Extended Data Fig. 7). In addition, scavenging by invertebrates and vertebrates is another factor that can affect not only the decomposer microbial composition (for example, carrion beetles)^26^ but also the microbes themselves which can shape the scavenger community via volatile organic compounds (for example, repel vertebrates but attract insects^48,66^). A better understanding of which intrinsic and extrinsic factors directly affect microbes that are important features for predicting PMI will be an important next step.

Our improved understanding of the microbial ecology of decomposing human cadavers and its more general implications for the crucial and rarely studied carrion nutrient pool is critical for revising concepts of what should be included in carbon and nutrient budgets and the models used to forecast ecosystem function and change^11^. New insight on the role of carrion decomposition in fuelling carbon and nutrient cycling is needed for conceptual and numerical models of biogeochemical cycles and trophic processes^11^; this study informs how the assembly and interactions among decomposer microbial communities facilitate the turnover and exchange of resources, and begins unlocking one of the remaining black boxes of ecosystem ecology. Finally, these findings may contribute to society by providing potential for a new forensic tool and for potentially modulating decomposition processes in both agricultural and human death industries via the key microbial decomposers identified here.

## Methods

### Site and donor selection

Outdoor experiments on 36 human cadavers were conducted at three willed-body donation facilities: Colorado Mesa University Forensic Investigation Research Station (FIRS), Sam Houston State University Southeast Texas Applied Forensic Science (STAFS) Facility and University of Tennessee Anthropology Research Facility (ARF). Before the start of the project, a meeting was held at STAFS to demonstrate, discuss and agree on sampling protocols. The Institutional Review Board and the Protection of Human Subjects Committee either determined that review was not required or granted exempt status for donors at each respective facility since the proposed research does not involve human donors as defined by federal regulations. Three deceased human donors were placed supine and unclothed on the soil surface in the spring, summer, fall and winter over the years 2016 and 2017 at each facility (*N* = 36). Bodies were placed on soil with no known previous human decomposition. Before placement, STAFS performed minimal removal of vegetation including raking of leaves and removal of shrubbery, and bodies placed at STAFS were placed in cages made of 1 cm × 1 cm wire fences and wooden frames to prevent vertebrate scavenging. The ARF and FIRS did not remove vegetation or place bodies under cages as standard protocol. Furthermore, bodies were placed no closer than 2.5 m between sternum midpoints. Collection date for each donor can be found in the sample metadata, in addition to cause of death if known, initial condition, autopsy status, weight before placement, age in years if known, estimated age if not known, sex, donor storage type, days donor was stored, time since death to cooling and placement head direction (Supplementary Table 1). Donor weight was taken at time of intake at ARF and FIRS but is a self-reported measure either by the donor before death or a family member at STAFS. During daily sampling, daily ambient average temperature and humidity, TBS^27^, scavenging status and insect status were recorded if available or applicable. Human bodies were fully exposed to all weather elements and invertebrate scavengers. Inclusion criteria for the remains were specified before the start of the experiment and required that the remains were in the fresh stage of decomposition and had not been frozen (and not extensively cooled) or autopsied before placement at the facility.

### Decomposition metric calculations

The Köppen–Geiger climate classification system characterizes both the ARF and STAFS facilities as temperate without a dry season and hot summer (Cfa) and the FIRS facility as a cold semi-arid steppe (BSk)^23^. Average daily temperatures were collected from the National Centers for Environmental Information (NCEI) website (https://www.ncei.noaa.gov/) and monthly total precipitation accumulation over the course of the study was collected from the Weather Underground website (https://www.wunderground.com/) from local weather stations: Grand Junction Regional Airport Station, McGhee Tyson Airport Station and Easterwood Airport Station. Reference ^27^ TBS quantifies the degree to which decomposition has occurred in three main areas (head, trunk and limbs)^27^. The user assigned values to represent the progress of decomposition on the basis of visual assessment of the cadaver and added these values to generate a TBS at the time of sampling. A maximum score was assigned for each area when the cadaver has reached dry skeletal remains. ADD was estimated using the weather data provided by the NCEI. Degree day on the day of placement was not included, and a base temperature of 0 °C was used. ADD was calculated by adding together all average daily temperatures above 0 °C for all previous days of decomposition, as in ref. ^27^, and subtracting the base temperature of 0 °C.

### Sample collection and DNA extraction

We sampled the skin surface of the head and torso near the hip along with gravesoils (soils associated with decomposition) associated with each skin site over 21 d of decomposition. Control soil samples were taken of the same soil series and horizon that are not associated with body decomposition (known past or present) from areas within or just outside each facility. We collected swabs of 756 non-decomposition soil (controls), 756 gravesoil near the hip, 756 gravesoil near the face, 756 hip skin and 756 face skin samples (*N* = 3,780). All site samples (skin surface, gravesoil and control soil) were taken using sterile dual-tipped BD SWUBE applicator (REF 281130) swabs as described in ref. ^18^, and immediately frozen after each sampling event and kept frozen at −20 °C. Samples were shipped to CU Boulder or Colorado State University overnight on dry ice and immediately stored at −20 °C upon arrival and until DNA extraction. Skin and soil DNA was extracted from a single tip of the dual-tipped swabs using the PowerSoil DNA isolation kit 96-htp (MoBio Laboratories), according to standard EMP protocols (http://www.earthmicrobiome.org/).

### Amplicon library preparation and sequencing

Bacterial and archaeal communities were characterized using 16S rRNA gene regions while eukaryotic communities were characterized using 18S rRNA gene regions as universal markers, for all successful skin and soil DNA extracts (*n* = 3,547). To survey bacteria and archaea, we used the primer set 515f (5′GTGYCAGCMGCCGCGGTAA) and 806rb (5′GGACTACNVGGGTWTCTAAT) that targets these domains near-universally^67,68^, with barcoded primers allowing for multiplexing, following EMP protocols^69^. To survey microbial eukaryotes, we sequenced a subregion of the 18S rRNA gene using the primers 1391f_illumina (5′GTACACACCGCCCGTC) and EukBr_illumina (5′TGATCCTTCTGCAGGTTCACCTAC) targeting the 3′ end of the 18S rRNA gene. 18S rRNA gene primers were adapted from ref. ^70^ and target a broad range of eukaryotic lineages. We have successfully generated and analysed data using these gene markers previously^6,18^. Primers included error-corrected Golay barcodes to allow for multiplexing while preventing misassignment. PCR amplicons were quantified using Picogreen Quant-iT (Invitrogen, Life Technologies) and pooled from each sample to equimolar ratio in a single tube before shipping to the UC San Diego genomics laboratory for sequencing. For both amplicon types, pools were purified using the UltraClean PCR clean-up kit (Qiagen). 16S rRNA pools were sequenced using a 300-cycle kit on the Illumina MiSeq sequencing platform and 18S rRNA gene pools were sequenced using a 300-cycle kit on the Illumina HiSeq 2500 sequencing platform (Illumina). Samples within a sample type (skin vs soil) were randomly assigned to a sequencing run to prevent potential batch effects. Blank DNA extraction and PCR negative controls were included throughout the entire process from DNA extraction to PCR amplification to monitor contamination (*n* = 592 negative controls).

### Shotgun metagenomic library preparation and sequencing

Extracted DNA from a subset of hip-associated soil samples (*n* = 756), soil controls (*n* = 9), blank controls (*n* = 102) and no-template PCR controls (*n* = 15) were chosen to undergo shallow shotgun sequencing to provide in-depth investigation of microbial dynamics within decomposition soil (Supplementary Table 4). Our standard protocol followed that of ref. ^71^ and was optimized for an input quantity of 1 ng DNA per reaction. Before library preparation, input DNA was transferred to 384-well plates and quantified using a PicoGreen fluorescence assay (ThermoFisher). Input DNA was then normalized to 1 ng in a volume of 3.5 μl of molecular-grade water using an Echo 550 acoustic liquid-handling robot (Labcyte). Enzyme mixes for fragmentation, end repair and A-tailing, ligation and PCR were prepared and added at 1:8 scale volume using a Mosquito HV micropipetting robot (TTP Labtech). Fragmentation was performed at 37 °C for 20 min, followed by end repair and A-tailing at 65 °C for 30 min. Sequencing adapters and barcode indices were added in two steps, following the iTru adapter protocol^72^. Universal adapter ‘stub’ adapter molecules and ligase mix were first added to the end-repaired DNA using the Mosquito HV robot and ligation performed at 20 °C for 1 h. Unligated adapters and adapter dimers were then removed using AMPure XP magnetic beads and a BlueCat purification robot (BlueCat Bio). A 7.5 μl magnetic bead solution was added to the total adapter-ligated sample volume, washed twice with 70% ethanol and then resuspended in 7 μl molecular-grade water.

Next, individual i7 and i5 indices were added to the adapter-ligated samples using the Echo 550 robot. Because this liquid handler individually addresses wells and we used the full set of 384 unique error-correcting i7 and i5 indices, we generated each plate of 384 libraries without repeating any barcodes, eliminating the problem of sequence misassignment due to barcode swapping (61, 62). To ensure that libraries generated on different plates could be pooled if necessary and to safeguard against the possibility of contamination due to sample carryover between runs, we also iterated the assignment of i7 to i5 indices per run, such that each unique i7:i5 index combination is only repeated once every 147,456 libraries^72^. A volume of 4.5 μl of eluted bead-washed ligated samples was added to 5.5 μl of PCR master mix and PCR-amplified for 15 cycles. The amplified and indexed libraries were then purified again using AMPure XP magnetic beads and the BlueCat robot, resuspended in 10 μl of water and 9 μl of final purified library transferred to a 384-well plate using the Mosquito HTS liquid-handling robot for library quantitation, sequencing and storage. All samples were then normalized on the basis of a PicoGreen fluorescence assay for sequencing.

Samples were originally sequenced on an Illumina HiSeq 4000; however, due to some sequencing failures, samples were resequenced on the Illumina NovaSeq 6000 platform. To ensure that we obtained the best sequencing results possible, we assessed both sequencing runs and added the best-performing sample of the two runs to the final analysis (that is, if sample X provided more reads from the HiSeq run than the NovaSeq run, we added the HiSeq data from that sample to the final analysis and vice versa). Samples were visually assessed to ensure that no batch effects from the two sequencing runs were present in beta diversity analysis. A list of which samples were pulled from the HiSeq vs NovaSeq runs can be found in the sample metadata under the column ‘best_MetaG_run’, with their corresponding read count under ‘MetaG_read_count’ (Supplementary Table 1). In total, 762 samples were sequenced, with 25 coming from the HiSeq run and 737 samples coming from the Novaseq run. Raw metagenomic data had adapters removed and were quality filtered using Atropos (v.1.1.24)^73^ with cut-offs of *q* = 15 and minimum length of 100 nt. All human sequence data were filtered out by aligning against the Genome Reference Consortium Human Build 38 patch release 7 (GRCh37/hg19) reference database released in 21 March 2016 (ncbi.nlm.nih.gov/assembly/GCF_000001405.13/) and removing all data that matched the reference from the sequence data. Alignment was performed with bowtie2 (v.2.2.3)^74^ using the --very-sensitive parameter, and the resulting SAM files were converted to FASTQ format with samtools (v.1.3.1)^75^ and bedtools (v.2.26.0)^76^. Metagenomic samples were removed from the analysis if they had <500 k reads. Final metagenomic sample numbers were 569 hip-adjacent soil, 5 soil controls, 102 blank controls and 15 no-template controls.

### Metabolite extraction and LC–MS/MS data generation

To investigate the metabolite pools associated with decomposition skin and gravesoils, we performed metabolite extraction on the second tip of the dual-tipped swabs collected from the skin and soil associated with the hip sampling location to ensure all datasets are paired. Skin and soil swab samples were extracted using a solution of 80% methanol. Briefly (with all steps performed on ice), swabs were placed into a pre-labelled 96-well DeepWell plate where A1–D1 were used for a solvent blank and E1–H1 were used for blank clean swabs with extraction solvent added. Swab shafts were cut aseptically and 500 μl of solvent (80% methanol with 0.5 μM sulfamethazine) was added. The DeepWell plate was covered and vortexed for 2 min, followed by 15 min in a water sonication bath. Next, samples were incubated at 4 °C for 2 h, followed by a 12 h incubation at −20 °C. Swab tips were then removed from the solvent and samples were lyophilised. Untargeted metabolomics LC–MS/MS data were generated from each sample. Two types of dataset were generated from each sample: MS1 data for global and statistical analysis and MS/MS data for molecular annotation. Molecular annotation was performed through the GNPS platform https://gnps.ucsd.edu/. Molecules were annotated with the GNPS reference libraries^77^ using accurate parent mass and MS/MS fragmentation pattern according to level 2 or 3 of annotation defined by the 2007 metabolomics standards initiative^78^. If needed and if the authentic chemical standard was available, MS/MS data were collected from the chemical standard and compared to MS/MS spectra of the molecule annotated from the sample (level 1 of annotation).

### Amplicon data processing

After data generation, amplicon sequence data were analysed in the Metcalf lab at Colorado State University using the QIIME2 analysis platform v.2020.2 and v.2020.8 (ref. ^79^). In total, 4,139 samples were sequenced, including 592 DNA extraction blank negative and no-template PCR controls. Sequencing resulted in a total of 89,288,561 16S rRNA partial gene reads and 1,543,472,127 18S rRNA partial gene reads. Sequences were quality filtered and demultiplexed using the pre-assigned Golay barcodes. Reads were 150 bp in length. 18S rRNA gene sequences had primers (5′GTAGGTGAACCTGCAGAAGGATCA) removed using cutadapt to ensure that the variable length of the 18S region was processed without primer contamination. Sequences were then classified into amplicon sequence variants (ASVs) in groups of samples that were included on the same sequencing run so the programme could accurately apply the potential error rates from the machine using the Deblur denoising method (v.2020.8.0)^80^. Feature tables and representative sequences obtained from denoising each sequencing run were then merged to create a complete dataset for each amplicon method. Taxonomic identifiers were assigned to the ASVs using the QIIME feature-classifier classify-sklearn method^81^. For the 16S rRNA gene data, these assignments were made using the SILVA 132 99% classifier for the 515fb/806rb gene sequences. ASVs that were assigned to chloroplast or mitochondria (non-microbial sequences) were filtered out of the dataset before continuing analysis. For 18S rRNA data, the RESCRIPt (v.2022.8.0) plugin was used to extract the full 12-level taxonomy from sequences matching the primers from the SILVA 138 99% database, to dereplicate the extracted sequences and to train a classifier to assign labels to ASVs in the feature table^82^. This taxonomy was used to filter out any ASVs that were assigned to Archaea, Streptophyta, Bacteria, Archaeplastida, Arthropoda, Chordata, Mollusca and Mammalia, as well as those that were unassigned, resulting in 5,535 ASVs at a total frequency of 772,483,701. DNA extraction negative and no-template PCR control samples were analysed to determine that contamination within the samples was minimal. Most control samples were low abundance and below the threshold used for rarefaction. The few controls that were above the rarefaction threshold clustered distantly and separately from true samples on principal coordinate analysis (PCoA) and had low alpha diversities, hence samples above the rarefaction depth were considered minimally contaminated and acceptable for analyses. Subsequently, DNA extraction negative and no-template PCR control samples were removed from the dataset and future analyses.

Microbial diversity metrics were generated from both amplicon types using the QIIME2 phylogenetic diversity plugin. The phylogenetic trees were constructed for each amplicon type individually using the fragment-insertion SEPP method^83^ against the SILVA 128 99% reference tree. Alpha diversity metrics were calculated using the number of observed features as ASV richness and Faith’s phylogenetic diversity formulas. Statistical comparisons were made using the pairwise Kruskal–Wallis *H*-test with a Benjamini–Hochberg multiple-testing correction at an alpha level of 0.05 (ref. ^84^). To evaluate beta diversity, the generalized UniFrac method weighted at 0.5 was used to calculate dissimilarity^85^. Statistical comparisons were made using permutational analysis of variance (PERMANOVA) with a multiple-testing correction and an alpha level of 0.05 (ref. ^86^). Taxonomy and alpha diversity visualizations were created using ggplot2 and the viridis package in R^87,88^. Beta diversity principal coordinates plots were constructed using the Emperor (v.2022.8.0) plugin in QIIME2 (ref. ^89^). Linear mixed-effects models were used to evaluate the contribution of covariates to a single dependent variable and to test whether community alpha diversity metrics (for example, ASV richness) and beta diversity distances (for example, UniFrac distances) were impacted by decomposition time (that is, ADD) and sampling location (that is, decomposition soil adjacent to the hip and control soil). The response variables were statistically assessed over ADD with sampling site (that is, decomposition soil vs control soil) as an independent variable (fixed effect) and a random intercept for individual bodies to account for repeated measures using the formula: diversity metric ≈ ADD × sampling site + (1|body ID).

### Detection of key decomposers in other decomposition studies

16S rRNA gene amplicon sequence data files from refs. ^6,24,25,64,69,90,91^ were obtained from QIITA^92^ under study IDs 10141–10143, 1609, 13114, 10317, 13301 and 11204, respectively. Data obtained from QIITA^92^ had been previously demultiplexed and denoised using Deblur^80^ and are available on the QIITA^92^ study page. Data from ref. ^16^ were obtained from the NCBI Sequence Read Archive under BioProject PRJNA525153. Forward reads were imported into QIIME2 (v.2023.5)^79^, demultiplexed and denoised using Deblur (v.1.1.1)^80^. Data from ref. ^26^ were obtained from the Max Planck Society Edmond repository (https://edmond.mpdl.mpg.de/dataset.xhtml?persistentId=doi:10.17617/3.UV4FBN). Forward reads were imported into QIIME2 (v.2023.5)^79^ and demultiplexed. Primers (5′ GTGCCAGCMGCCGCGGTAA) were removed using cutadapt (v.4.4)^93^ and the data were denoised using Deblur (v.1.1.1)^80^. ASVs from all studies were assigned taxonomy using a naïve Bayes taxonomy classifier trained on the V4 (515f/806r) region of SILVA 138 99% operational taxonomic units (OTUs). Data tables were imported into Jupyter notebooks (Jupyter Lab v.4.0.5)^94^ for further analysis (Python v.3.8.16). A search for the 35 universal PMI decomposer ASVs was conducted within each dataset. This search matched exact ASVs in our dataset to other datasets but did not match similar ASVs that may be classified as the same taxon. The relative abundance of each decomposer ASV was first averaged across all samples within a specific metadata category. The average relative abundances were then summed across each decomposer genus. Prevalence tables were constructed by summing the number of samples across a specific metadata category in which each universal decomposer ASV was present. The presence of *Wohlfahrtiimonas* was found in the ref. ^26^ dataset; however, these ASVs were not exact sequence matches to our universal *Wohlfahrtiimonas* decomposers and probably represent insect-associated strains (Supplementary Table 33; Wohlfahrtiimonadaceae column). We searched within the remaining studies for the presence of other ASVs assigned to the *Wohlfahrtiimonas* genus or ASVs that were assigned to the Wohlfahrtiimonadaceae family but these were unidentified at the genus level. Average relative abundances were calculated as described above.

### Community assembly mechanism determination

To investigate the ecological processes driving bacterial assembly, we quantitatively inferred community assembly mechanisms by phylogenetic bin-based null model analysis of 16S rRNA gene amplicon data as described in refs. ^95,96^. Longitudinal turnover in phylogenetic composition within the decomposition soil between successional stages was quantified using the beta nearest taxon index (βNTI), where a |βNTI| value <+2 indicates that stochastic forces drive community assembly and a value >+2 indicates less than or greater than expected phylogenetic turnover by random chance (deterministic forces). βNTI values <−2 correspond to homogeneous selection and values >+2 correspond to heterogeneous selection. Homogeneous selection refers to communities that are more similar to each other than expected by random chance, while heterogeneous selection refers to communities that are less similar to each other than expected by random chance. Deterministic forces include selection factors such as environmental filtering and biological interactions, while stochastic forces include random factors such as dispersal, birth–death events and immigration.

### MAGs generation and classification

To maximize assembly, metagenomes were co-assembled within sites using MEGAHIT (v.1.2.9)^97^ with the following flags: –k-min 41 (see Supplementary Tables 4–6 for a list of samples used to generate metagenomic data, co-assembly statistics, GTDB taxonomic classification and TPM-normalized count abundance of MAGs within each sample). Assembled scaffolds >2,500 kb were binned into MAGs using MetaBAT2 (v.2.12.1)^98^ with default parameters. MAG completion and contamination were assessed using checkM (v.1.1.2)^99^. MAGs were conservatively kept in the local MAG database if they were >50% complete and <10% contaminated. MAGs were dereplicated at 99% identity using dRep (v.2.6.2)^100^. MAG taxonomy was assigned using GTDB-tk (v.2.0.0, r207)^101^. Novel taxonomies were determined as the first un-named taxonomic level in the GTDB classification string (see Supplementary Table 5 for MAG quality and taxonomy information). MAGs and co-assemblies were annotated using DRAM (v.1.0.0)^102^ (Supplementary Table 5;10.5281/zenodo.7843104). From 575 metagenomes, we recovered 1,130 MAGs, of which 276 were medium or high quality, and dereplicated these at 99% identity into 257 MAGs. This MAG set encompassed novel bacterial orders (*n* = 3), families (*n* = 9), genera (*n* = 28) and species (*n* = 158), providing genomic blueprints for microbial decomposers dominated by Gammaproteobacteria and Actinobacteriota (Supplementary Table 5).

### MAG and gene abundance mapping

**Extended Data Fig. 10:**
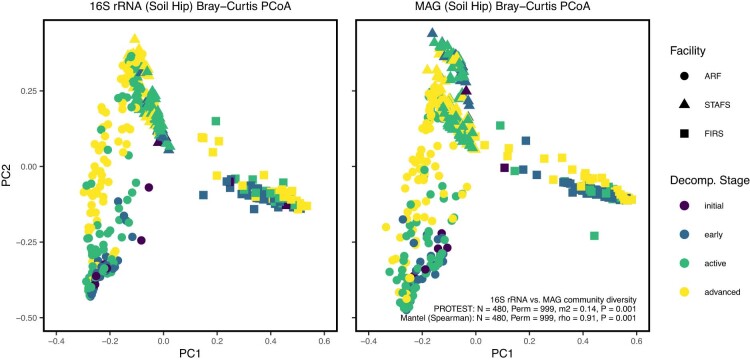
Diversity Comparison between 16S rRNA and Metagenomic Data. PCoA ordination plots of Bray-Curtis dissimilarity matrices calculated from paired rarefied 16S rRNA feature abundances (left) and TPM-normalized MAG abundances (right) from the soil adjacent to the hip. Procrustes/PROTEST and mantel tests were performed between the PCoA ordinances and distance matrices, respectively. n = 480 biologically independent samples, respectively.

To determine the abundance of the MAGs in each sample, we mapped reads from each sample to the dereplicated MAG set using bowtie2 (v.2.3.5)^74^ with the following flags: -D 10 -R 2 -N 1 -L 22 -i S,0,2.50. Output sam files were converted to sorted BAM files using samtools (v.1.9)^75^. BAM files were filtered for reads mapping at 95% identity using the reformat.sh script with flag idfilter=0.95 from BBMap (v.38.90) (https://sourceforge.net/projects/bbmap/). Filtered BAM files were input to CoverM (v0.3.2) (https://github.com/wwood/CoverM) in genome mode to output transcripts per million (TPM). To determine the abundance of genes across samples, we clustered the gene nucleotide sequences from the annotated assemblies output by DRAM using MMseqs2 (release 13) easy-linclust (v4e23d5f1d13a435c7b6c9406137ed68ce297e0fc)^103^ with the following flags: –min-seq-id 0.95–alignment-mode 3–max-seqs 100000. We then mapped reads to the cluster representative using bowtie2 (ref. ^74^) and filtered them to 95% identity as described above for the MAGs. To determine gene abundance, filtered bams were input to coverM in contig mode to output TPM. Bacterial MAG feature tables were imported into QIIME2 (v.2020.8)^79^. Bacterial features that were not present for a total of 50 times and were found in less than six samples were removed from the dataset to reduce noise. Bacterial feature tables were collapsed at the phylum, class, order, family, genus and species GTDB taxonomic levels. Community diversity was compared between the MAG and 16S rRNA ASV feature tables to ensure that both data types demonstrate the same biological signal. Each table was filtered to contain samples with paired 16S rRNA and metagenomic data (that is, samples with both metagenomic and 16S rRNA data). Bray–Curtis dissimilarity matrices were calculated for the TPM-normalized MAG abundance table and rarified 16S rRNA ASV table. Procrustes/PROTEST^104,105^ and Mantel tests were performed between the PCoA ordinations and distance matrices, respectively^106^. Results showed that the datasets were not significantly different from each other and confirmed their shared biological signal (Extended Data Fig. 10).

### Metabolic interaction simulations

Higher-order (20 microbial members) co-occurrence patterns were calculated from the MAG relative frequency tables of each decomposition stage (that is, early, active, advanced) for each facility using HiOrCo (v.1.0.0) (cut-off 0.001) (https://github.com/cdanielmachado/HiOrCo). HiOrCo provides 100 iterations of co-occurring MAG communities to improve simulation accuracy. No significantly co-occurring MAGs were detected at the FIRS facility during advanced decomposition; therefore, we continued the analyses using only early and active decomposition stages at FIRS. CarveMe (v.1.5.1)^107^ was used to construct genome-scale metabolic models (GEMs) from each MAG using default parameters (https://github.com/cdanielmachado/carveme). GEMs from each co-occurring MAG community were input as a microbial community into SMETANA (v1.0.0) (https://github.com/cdanielmachado/smetana) to compute several metrics that describe the potential for metabolic cooperative and competitive interactions between community members as described in refs. ^34,35^. Metrics include metabolic interaction potential (MIP), metabolic resource overlap (MRO), species coupling score (SCS), metabolite uptake score (MUS), metabolite production score (MPS) and SMETANA score. MIP calculates how many metabolites the species can share to decrease their dependency on external resources. MRO is a method of assessing metabolic competition by measuring the overlap between the minimal nutritional requirements of all member species on the basis of their genomes. SCS is a community size-dependent measurement of the dependency of one species in the presence of the others to survive. MUS measures how frequently a species needs to uptake a metabolite to survive. MPS is a binary measurement of the ability of a species to produce a metabolite. The individual SMETANA score is a combination of the SCS, MUS and MPS scores and gives a measure of certainty of a cross-feeding interaction (for example, species A receives metabolite X from species B). Simulations were created on the basis of a minimal medium, calculated using molecular weights, that supports the growth of both organisms, with the inorganic compounds hydrogen, water and phosphate excluded from analysis. A random null model analysis was performed to ensure that changes in co-occurring MAGs within each site and decomposition are driving interaction potential changes. For each site and decomposition stage, 100 20-member communities were generated by random selection without replacement using random.sample(). Simulations to calculate MIP and MRO were performed as above. A detailed investigation into the potential molecules being cross-fed was performed on the late stages of decomposition for each facility: temperate-climate advanced decomposition and semi-arid active decomposition stages.

### Metabolic efficiency simulations

Metabolic models and the Constraint Based Reconstruction and Analysis (COBRA) toolbox (v.3.0)^108^ were used to simulate differences in metabolic capabilities between samples that are spatiotemporally different. A general base growth medium, M_0_, containing a list of carbohydrates, amino acids, lipids and other vitamins and minerals adapted from a previous study^109^ was used. From this base medium, carbohydrate-rich, M_1_, amino acid-rich, M_2_, and lipid-rich, M_3_, media were defined. The carbohydrate-rich medium includes all compounds in the base medium but allows for higher uptake of carbohydrates than proteins and lipids, and vice versa. The COBRA toolbox^108^ in MATLAB was used to optimize overall ATP production from M_1_, M_2_ and M_3_ for each individual MAG in an aerobic condition. This assumption was made because the topsoil conditions in which decomposition happens are relatively aerobic. The calculated maximum ATP yields can be interpreted as the maximum capability of each MAG in extracting ATP from the growth media. Finally, the weighted average of total ATP production from the GEMs in a sample was calculated by multiplying the relative abundance of each MAG by the maximum total ATP production and summing over all of the GEMs in a sample^110^.

### Molecular networking and spectral library search

A molecular network was created using the Feature-Based Molecular Networking (FBMN) workflow (v.28.2)^111^ on GNPS (https://gnps.ucsd.edu; ref. ^77^). The mass spectrometry data were first processed with MZMINE2 (v.2.53)^112^ and the results were exported to GNPS for FBMN analysis. The precursor ion mass tolerance was set to 0.05 Da and the MS/MS fragment ion tolerance to 0.05 Da. A molecular network was then created where edges were filtered to have a cosine score above 0.7 and >5 matched peaks. Furthermore, edges between two nodes were kept in the network if and only if each of the nodes appeared in each other’s respective top 10 most similar nodes. Finally, the maximum size of a molecular family was set to 100, and the lowest-scoring edges were removed from molecular families until the molecular family size was below this threshold. The spectra in the network were then searched against GNPS spectral libraries^77,111^. All matches kept between network spectra and library spectra were required to have a score above 0.7 and at least 6 matched peaks.

### Metabolite formula and class prediction

Spectra were downloaded from GNPS and imported to SIRIUS (v.4.4)^113^ containing ZODIAC^114^ for database-independent molecular formula annotation under default parameters. Formula annotations were kept if the ZODIAC score was at least 0.95 and at least 90% of the MS/MS spectrum intensity was explained by SIRIUS as described by the less-restrictive filtering from ref. ^114^. A final list of formula identifications was created by merging ZODIAC identifications with library hits from GNPS (Supplementary Table 36). In the cases where a metabolite had both a ZODIAC predicted formula and an assigned library hit, the library hit assignment took precedence. The final formula list contained 604 formula assignments. Organic compound composition was examined in van Krevelen diagrams and assigned to major biochemical classes on the basis of the molar H:C and O:C ratios^115^. Since classification based on molecular ratio does not guarantee that the compound is part of a specific biochemical class, compounds were labelled as chemically similar by adding ‘-like’ to their assigned class (for example, protein-like). Furthermore, compound formulas were used to calculate the nominal oxidation state of carbon on the basis of the molecular abundances of C, H, N, O, P and S as described in ref. ^116^ (Supplementary Tables 37 and 38).

### Metabolite feature table processing

The metabolite feature table downloaded from GNPS was normalized using sum normalization, then scaled with pareto scaling^117^ and imported in QIIME2 (v.2022.2)^79^. This table contains all library hits, metabolites with predicted formulas and unannotated metabolites. PCoA clustering with Bray–Curtis and Jaccard distances confirmed clustering of processing controls separate from soil and skin samples. Five soil samples were removed for clustering with processing controls. Processing controls were removed from the dataset; then metabolites absent from a minimum of 30 samples were removed to reduce noise. Bray–Curtis and Jaccard beta diversity group comparisons were performed between soil and skin samples using PERMANOVA (perm. = 999). The metabolite feature table was filtered to contain metabolites with chemical formulas based on GNPS library hits and/or predicted chemical formulas from ZODIAC. Differential abundance analyses were performed on these tables from the cadaver-associated soil and skin to test metabolite log-ratio change over decomposition stage using initial, day 0 samples as the reference frame, utilizing the Analysis of Composition of Microbiomes with Bias Correction (ANCOM-BC)^118^ QIIME2 (v.2022.2) plugin.

### Joint-RPCA

The complete methodology including mathematical formulas for joint-RPCA can be found in Supplementary Text. Briefly, before joint factorization, we first split the dataset into training train and testing sample sets from the total set of shared samples across all input data matrices. The datasets included in this analysis were 16S rRNA gene abundances, 18S rRNA gene abundances, MAG abundances, MAG gene abundances, MAG gene functional modules and metabolites from the hip-adjacent decomposition soil. Each matrix was then transformed through the robust-centred-log-ratio transformation (robust-clr) to centre the data around zero and approximate a normal distribution^42,119^. Unlike the traditional clr transformation, the robust-clr handles the sparsity often found in biological data without requiring imputation. The robust-clr transformation was applied to the training and test set matrices independently. The joint factorization used here was built on the OptSpace matrix completion algorithm, which is a singular value decomposition optimized on a local manifold^42,119^. A shared matrix was estimated across the shared samples of all input matrices. For each matrix, the observed values were only computed on the non-zero entries and then averaged, such that the minimized shared estimated matrices were optimized across all matrices. The minimization was performed across iterations by gradient descent. To ensure that the rotation of the estimated matrices was consistent, the estimated shared matrix and the matrix of shared eigenvalues across all input matrices were recalculated at each iteration. To prevent overfitting of the joint-factorization, cross-validation of the reconstruction was performed. In this case, all the previously described minimization was performed on only the training set data. The test set data were then projected into the same space using the training set data estimated matrices and the reconstruction of the test data was calculated. Through this, it can be ensured that the minimization error of the training data estimations also minimizes that of the test set data, which is not incorporated into these estimates on each iteration. After the training data estimates were finalized, the test set samples were again projected into the final output to prevent these samples from being lost. The correlations of all features across all input matrices were calculated from the final estimated matrices. Finally, here we treated the joint-RPCA with only one input matrix as the original RPCA^119^ but with the additional benefit of the addition of cross-validation for comparison across other methods.

### Multi-omics ecological network visualization

The datasets included in this analysis were 18S rRNA gene abundances, MAG abundances, MAG gene functional modules and metabolites from the hip-adjacent decomposition soil. log ratios were generated using the joint-RPCA PC2 scores, chosen on the basis of the sample ordination, to rank each omics feature on the basis of association with either initial non-decomposition and early decomposition soil or late decomposition (that is, active and advanced) soil time periods. The log ratios are the log ratio of the sum of the top *N*-features raw-counts/table-values over the sum of the bottom *N* ranked features raw-counts/table-values, based on the PC2 loadings produced from the ordinal analysis since these were observed to change the most by decomposition stage. To prevent sample drop out in the log ratio due to sparsity, as described in refs. ^120,121^, between 2 and 1,500 numerator and denominator features for each omic were summed such that at least 90% of the sample were retained: metagenomics (MAGs) *N*-features = 30 (99.2%), 18S *N*-features = 1,499 (90.1%), metagenomics (gene modules) *N*-features = 26 (100%) and metabolomics *N*-features = 238 (100%). The joint-RPCA correlation matrix was subset down to the total initial day zero, early, active or advanced decomposition-associated features used in the log ratios to generate the network visualizations. Only the top 20% of correlations between selected nodes were retained to reduce noise in generating the network visualization.

### Phylogenetic tree generation

Redbiom (v.0.3.9)^122^ was used to search for all publicly available AGP^90^ and EMP^69^ studies for samples containing at least 100 counts of a key decomposer. The AGP samples were further filtered to only include gut and skin environments and the EMP samples were limited to only include soil and host environment. Next, the top 50 most abundant ASVs were taken from each environment along with the key decomposers and placed on a phylogenetic tree using Greengenes2 (release 2022.10)^123^. The ASVs were then ranked according to the number of samples they were found in and visualized using EMPress (v.1.2.0)^124^.

### Random forest regression modelling

Processed features tables from each ‘omic data type were used for random forest regression modelling with nested cross-validation (CV) to test ADD prediction power. Data were subset so that models were trained and tested for each sampling location separately (for example, soil adjacent to the hip, soil adjacent to the face, skin of the hip and skin of the face). Data were pre-processed for models using calour (v.2018.5.1) (http://biocore.github.io/calour/index.html) and models were trained/tested using scikit-learn (v.0.24.2)^125^. Features with an abundance of zero in the dataset after filtering were removed. The facilities at which sampling was performed were included as features in the model to determine whether geographical location is important for modelling. Samples from individual bodies were grouped together to prevent samples from a body being split between train and test sets to help prevent overfitting. Nested CV was performed to thoroughly test the accuracy and generalizability of the models. Hyperparameters tested for optimization were: max_depth = [None, 4], max_features = [‘auto’, 0.2] and bootstrap = [True, False]. Nested CV was made of an outer CV loop and an inner CV loop. The outer loop was created by a LeaveOneGroupOut split wherein samples from one of the 36 bodies were set aside for model validation after the inner CV loop completes. The remaining 35 bodies were used for RandomForestRegressor (n_estimators = 500) model training with the inner CV loop. The inner CV loop performed a LeaveOneGroupOut split as well so that 34 bodies were used to train a model, which was tested on the samples from the one withheld body in the inner CV loop. This inner CV was repeated until all 35 bodies within the inner loop were used as a test body once to determine which hyperparameters were best for prediction. The best-performing inner CV model was then used to predict the samples from the 36th body that was withheld at the outer CV loop, which now acts as a validation test set. Model accuracy was determined by calculating the MAE of the predicted ADD relative to the actual ADD of all the validation body samples. The prediction of the samples from the 36th body, which was completely withheld from the training of the model, allowed us to reduce overfitting and gain an estimate of the model accuracy. The entire nested CV process was repeated until each body was used as the outer CV loop validation body one time (that is, 36 iterations). The resulting 36 mean absolute errors of each body were used for determining model accuracy, generalizability and which data type performed the best. To ensure that we were using the complete dataset to determine the important taxa driving the models, the best-performing hyperparameters (bootstrap=False, max_depth=None, max_features=0.2) were used to train a RandomForestRegressor (n_estimators = 1,000) model to extract the important features. Important features were ranked by their relative importance on a scale from 0–1, where the sum of all importances equals 1. A random forest model using TBS from each sampling day as training data for ADD prediction was trained and tested using the same methodology to compare microbiome-based models to a more traditional method of assessing decomposition progression.

Lastly, we confirmed the accuracy and reliability of postmortem interval prediction with an independent test set of samples collected from bodies not represented in our models. The independent test set was collected from hip-adjacent soil and skin of the hip locations across three facilities (ARF, Forensic Anthropology Research Facility in San Marcos, Texas (FARF) and Research on Experimental and Social Thanatology in Quebec, Canada (REST)) (Supplementary Table 39). The independent test set was made up of temporal samples taken from each facility. ARF and REST samples consisted of three bodies with three timepoints taken from each body at each facility. At each timepoint, a soil sample was swabbed within the purge and outside the purge, and a skin sample was swabbed from the hip. One ARF body (B3.D4) did not have purge during the first timepoint; therefore, this sample was not collected. FARF provided samples from four bodies. Two bodies (2021.04 and 2021.45) had the same sampling procedure as ARF and REST, while the other two bodies (2021.39 and 2021.44) did not have purge during the first sampling timepoint; hence samples were not collected. Samples were collected, shipped, stored, DNA extracted and 16S rRNA V4 sequenced using the previously described methods. After data generation, amplicon sequence data were analysed in the Metcalf lab using QIIME2 (v.2020.8)^79^. Sequences were quality filtered and demultiplexed using the pre-assigned Golay barcodes. Reads were 150 bp in length. Sequences were then classified into ASVs using the deblur denoising method^80^. Taxonomic identifiers were assigned to the ASVs using the QIIME feature-classifier classify-sklearn method^81^ using the SILVA 132 99% classifier for the 515fb/806rb gene sequences. ASVs that were assigned to chloroplast or mitochondria (non-microbial sequences) were filtered out of the dataset before continuing analysis. Data were rarified to 5,000 reads per sample and collapsed to the SILVA database 7-rank taxonomic level (L7). Feature tables were split into soil and skin data; then the validation data table was matched to the original dataset so that sampling location and features were the same (that is, using only taxa found in hip-adjacent soil in both datasets). A random forest regressor model (n_estimators=1000, max_depth=None, bootstrap=False, max_features=0.2) was built and fitted to predict the validation samples’ true ADD measurement. Randomly assigned ADDs were used as a null model.

### Statistics and reproducibility

From March 2016 to December 2017, 36 human cadavers were sampled daily starting on the day of placement through 21 d of decomposition. The study encompasses three geographically distinct anthropological research facilities, and 3 cadavers were placed at each facility for each of the four seasons. Swab samples were collected from soil directly adjacent to the hip, face and a control, non-decomposition location. Swab samples were also collected from skin located on the hip and the face. No statistical method was used to predetermine sample size. The samples were randomized during processing. The investigators were not blinded to allocation during experiments and outcome assessment. Samples were excluded if not enough DNA was extracted, sequenced or if sequence quality was poor. Negative controls were included during DNA/metabolite extraction, amplification and library preparation. Linear statistical modelling was performed with linear mixed-effects models to a single dependent variable, and response variables were statistically assessed over ADD with a random intercept for individual bodies to account for repeated measures. Group comparisons were performed using Dunn Kruskal–Wallis *H*-test with multiple-comparison *P* values adjusted using the Benjamini–Hochberg method, two-tailed analysis of variance (ANOVA) with no multiple-comparison adjustments, or PERMANOVA with a multiple-testing correction. Differential abundance analyses were performed using ANCOM-BC^118^ with initial, day 0 samples as the reference frame. Procrustes/PROTEST^104,105^ and Mantel tests were performed between PCoA ordinations and distance matrices, respectively^106^.

### Reporting summary

Further information on research design is available in the Nature Portfolio Reporting Summary linked to this article.

### Supplementary information

Supplementary Information Legends for Supplementary Tables 1–9, 14–16 and 25–39. Supplementary Tables 10–13 and 17–24, and Text.

Reporting Summary

Supplementary Tables Supplementary Table 1. Sample metadata. Table includes data taken during intake and over the course of the study. Table 2. ANCOM-BC differential abundance analysis results of cadaver skin metabolite log-ratio change over decomposition stages. Initial day 0 samples were used as the reference level and the intercept. Results include log-ratio changes of day 0 metabolites to early, active and advanced decomposition stages, *P* values, Holm–Bonferroni-corrected *P* values (*Q* values), standard errors and *W* values. Table 3. ANCOM-BC differential abundance analysis results of cadaver-associated soil metabolite log-ratio change over decomposition stages. Initial day 0 samples were used as the reference level and the intercept. Results include log-ratio changes of day 0 metabolites to early, active and advanced decomposition stages, *P* values, Holm–Bonferroni-corrected *P* values (*Q* values), standard errors and *W* values. Table 4. List of samples used to generate shotgun metagenomic data. Table 5. Assembly statistics and GTDB taxonomic classification of genomic bins (metagenome-assembled genomes; MAGs) co-assembled from the metagenomic samples. Table includes completeness and contamination of each MAG. Table 6. TPM-normalized count abundance of MAGs within metagenomic samples. Table 7. Linear mixed-effects model statistics for testing response variable change of ATP per C-mol amino acids calculated from metagenomic data over ADD at each facility and a random intercept for each individual body to account for repeated measures to test whether the metabolism efficacy shifts within each facility. Formula: ‘ATPm ≈ ADD + (1|body ID)’. Table 8. Linear mixed-effects model statistics for testing response variable change of ATP per C-mol carbohydrates calculated from metagenomic data over ADD at each facility and a random intercept for each individual body to account for repeated measures to test whether the metabolism efficacy shifts within each facility. Formula: ‘ATPm ≈ ADD + (1|body ITable 9. Linear mixed-effects model statistics for testing response variable change of ATP per C-mol lipids calculated from metagenomic data over ADD at each facility and a random intercept for each individual body to account for repeated measures to test whether the metabolism efficacy shifts within each facility. Formula: ‘ATPm ≈ ADD + (1|body ID)’. Table 14. Number of predicted exchanges for cross-fed compounds at each facility during late decomposition. Late decomposition was defined as the advanced decomposition stage at STAFS and ARF and the active decomposition stage at FIRS. Table 15. Linear mixed-effects model statistics for testing response variable change of Generalized UniFrac PC1 distances calculated from 16S rRNA gene data over ADD at each facility with sampling site (that is, soil adjacent to hip vs soil control) as an independent variable (fixed effect) and a random intercept for each individual body to account for repeated measures. The models measure the sampling site and ADD variables individually and the interaction between the variables. The interaction between the variables was used to test whether the sampling sites respond differently to decomposition. Formula: ‘diversity metric ≈ ADD × sampling site + (1|body ID)’. Table 16. Linear mixed-effects model statistics for testing response variable change of ASV richness calculated from 16S rRNA gene data over ADD at each facility with sampling site (that is, soil adjacent to hip vs soil control) as an independent variable (fixed effect) and a random intercept for each individual body to account for repeated measures. The models measure the sampling site and ADD variables individually and the interaction between the variables. The interaction between the variables was used to test whether the sampling sites respond differently to decomposition. Formula: ‘diversity metric ≈ ADD × sampling site + (1|body ID)’. Table 25. Joint-RPCA PC2 correlations calculated between network feature nodes that correspond with late (that is, active and advanced) decomposition soil. Table 26. Joint-RPCA PC2 correlations calculated between network feature nodes in initial, non-decomposition and early decomposition soil. Table 27. 16S rRNA gene ASVs assigned to the same taxonomy as decomposer network taxa. Table includes the phylogenetic tree labels in Fig. 4e, 150-bp-long ASVs and trimmed 100-bp-long ASVs used to explore ASV presence in other studies. Table 28. Presence of universal decomposers in possible human and terrestrial source environments in a few other studies. Table shows the average relative abundance of each decomposer ASV across each sample type. Average relative abundances were then summed for each decomposer genus. Table 29. Cross-feeding statistics for MAGs predicted as cross-feeders during late decomposition. Table includes GTDB taxonomic classification, number of reactions each MAG was considered the compound receiver and/or donor, and the percent responsible for all donations and acceptances during late decomposition. Late decomposition was defined as the advanced decomposition stage at STAFS and ARF and the active decomposition stage at FIRS. Table 30. Cross-feeding exchanges for *Oblitimonas alkaliphila* during late decomposition. *Oblitimonas alkaliphila* was not a predicted cross-feeder at FIRS during this timeframe. Table includes MAG ID and taxonomic classification of genomes involved in exchange, compounds exchanged and computed interaction metrics. Table 31. Cross-feeding exchanges for l-arginine or ornithine during late decomposition. Table includes MAG ID and taxonomic classification of genomes involved in exchange, compounds exchanged and computed interaction metrics. Table 32. Model validation results from predicting an independent test set of samples using the 16S rRNA gene at the SILVA database level-7 taxonomic rank random forest regression models for the skin of the hip and soil adjacent to the hip. Errors are represented by MAE in ADD. Table 33. Presence of universal decomposers in a few other studies focused on mammalian decomposition environments. A search for the 35 universal PMI decomposer ASVs was conducted within each dataset. The relative abundance of each decomposer ASV was first averaged across all samples within a specific metadata category. The average relative abundances were then summed across each decomposer genus. Prevalence tables were constructed by summing the number of samples across a specific metadata category in which each universal decomposer ASV was present. Table 34. The average ADD per calendar day calculated for each cadaver at each facility. The average ADD per calendar day was calculated by dividing the final maximum ADD values by the total number of days (that is, 21). The average ADD per day was calculated for each cadaver, season and facility, each climate type and as a study-wide average. Table 35. The average ADD per calendar day calculated for each cadaver at each facility for the independent test set. The average ADD per calendar day was calculated by dividing the final maximum ADD values by the total number of sampling days. The average ADD per day was calculated for each cadaver, facility and as a study-wide average. Table 36. Metabolite identification information for metabolites that had a predicted chemical formula or matched to a compound in the database library. When available, chemical formulas in the database library took precedence over predicted chemical formulas for calculating NOSC and major biochemical classes based on the molar H:C and O:C ratios. Table 37. Soil metabolite feature table normalized with sum normalization then scaled with pareto scaling. Table includes chemical formulas and major biochemical classes based on the molar H:C and O:C ratios. Table 38. Skin metabolite feature table normalized with sum normalization then scaled with pareto scaling. Table includes chemical formulas and major biochemical classes based on the molar H:C and O:C ratios. Table 39. Sample metadata for the machine learning independent test set. Table includes data taken during intake and over the course of the study.

### Source data

Source Data for Figs. 1–6, Extended Data Figs. 1–6 and Extended Data Fig. 9 SD for Fig. 1. Sample type counts and sample metadata. SD for Fig. 2. ATP per C-mol for each substrate by sample and pairwise beta-NTI calculations. SD for Fig. 3. SMETANA MIP and MRO score calculations, predicted cross-fed metabolites, Faith’s PD calculations and joint-RPCA distance matrix/ordination. SD for Fig. 4. Joint-RPCA distance matrix/ordination and multi-omic log ratios. SD for Fig. 5. Late decomposition multi-omic correlations. SD for Fig. 6. Random forest predictions, 16S rRNA model important features and 16S rRNA SILVA-L7 feature table. SD for ED Fig. 1. Site weather data. SD for ED Fig. 2. Metabolite feature table, chemical formulas and Van Krevelen metabolite classifications. SD for ED Fig. 3. MAG taxonomy and feature table, amino acid and carbohydrate ATP per C-mol per MAG and sample. SD for ED Fig. 4. 16S rRNA calculated richness. SD for ED Fig. 5. Initial/early decomposition multi-omic correlations. SD for ED Fig. 6. Top rank taxa for phylogenetic tree comparing ASVs found during decomposition and in the EMP and AGP datasets. SD for ED Fig. 9. 16S rRNA random forest validation predictions

## Data Availability

Raw amplicon and metagenomic sequencing data and sample metadata are available on the QIITA open-source microbiome study management platform under study 14989 and ENA accession PRJEB62460 (ERP147550). Dereplicated MAGs and DRAM output can be found publicly on Zenodo (10.5281/zenodo.7843104; https://zenodo.org/record/7938240) and NCBI BioProject PRJNA973116. The mass spectrometry data were deposited on the MassIVE public repository (accession numbers: MSV000084322 for skin samples and MSV000084463 for soil samples). The molecular networking job can be publicly accessed at https://gnps.ucsd.edu/ProteoSAFe/status.jsp?task=1c73926f2eb5409985cc2e136062db2f. The GNPS database was accessed through https://gnps.ucsd.edu/. The GreenGenes2 database can be found at https://ftp.microbio.me/greengenes_release/. SILVA databases can be found at https://www.arb-silva.de/documentation/release-1381/. The Earth Microbiome Project data and American Gut Project data can be found on EBI under accessions ERP125879 and ERP012803, respectively. 16S rRNA gene amplicon sequence data files from refs. ^6,24,25,64,69,90,91^ were obtained from QIITA^92^ under study IDs 10141–10143 (ref. ^6^), 1609 (refs. ^24,25^), 13114 (ref. ^69^), 10317 (ref. ^90^), 13301 (ref. ^64^) and 11204 (ref. ^91^). Data from ref. ^16^ were obtained from the NCBI Sequence Read Archive under BioProject PRJNA525153. Data from ref. ^26^ were obtained from the Max Planck Society Edmond repository (https://edmond.mpdl.mpg.de/dataset.xhtml?persistentId=doi:10.17617/3.UV4FBN). The GTDB data can be accessed at https://data.gtdb.ecogenomic.org/releases/. Source data are provided with this paper.

## Code availability

Analysis code, intermediate files and metadata are publicly available on Github (https://github.com/Metcalf-Lab/2023-Universal-microbial-decomposer-network). The complete mathematical algorithms for Joint-RPCA can be found in Supplementary Text.
