# Supplementary material for: A conserved interdomain microbial network underpins cadaver decomposition despite environmental variables
Source: Nat Microbiol. 2024 Feb 12;9(3):595–613. doi: 10.1038/s41564-023-01580-y (PMC10914610; doi:10.1038/s41564-023-01580-y)
Supplement: Supplementary file 1 — Legends for Supplementary Tables 1–9, 14–16 and 25–39. Supplementary Tables 10–13 and 17–24, and Text. [file 41564_2023_1580_MOESM1_ESM.pdf]

nature microbiology

Article https://doi.org/10.1038/s41564-023-01580-y

A conserved interdomain microbial network underpins cadaver decomposition despite environmental variables

In the format provided by the authors and unedited

Supplementary Information for

A conserved interdomain microbial network underpins cadaver decomposition despite environmental variables

Zachary M. Burcham[1,2], Aeriel D. Belk[1,3], Bridget B. McGivern[4], Amina Bouslimani[5], Parsa Ghadermazi[6], Cameron Martino[7], Liat Shenhav[8,9,10], Anru R. Zhang[11,12], Pixu Shi[11], Alexandra Emmons[1], Heather Deel[1], Zhenjiang Zech Xu[13], Victoria Nieciecki[1,14], Qiyun Zhu[7,15,16], Michael Shaffer[4], Morgan Panitchpakdi[5], Kelly C. Weldon[5], Kalen Cantrell[17], Asa Ben-Hur[18], Sasha C. Reed[19], Greg C. Humphry[7], Gail Ackermann[7], Daniel McDonald[7], Siu Hung Joshua Chan[6], Melissa Connor[20], Derek Boyd[21,22], Jake Smith[21,23], Jenna M.S. Watson[21], Giovanna Vidoli[21], Dawnie Steadman[21], Aaron M. Lynne[24], Sibyl Bucheli[24], Pieter C. Dorrestein[5], Kelly C. Wrighton[4], David O. Carter[25], Rob Knight[7,17,26,27], Jessica L. Metcalf[1]*

Corresponding author: jessica.metcalf@colostate.edu

The PDF file includes:

Legends for Supplementary Tables 1 to 9, 14 to 16, and 25 to 39
Supplementary Tables 10 to 13 and 17 to 24
Supplementary Text

Other Supplementary Material for this manuscript includes the following:

Supplementary Tables 1 to 9, 14 to 16, and 25 to 39 (separate file)

Supplementary Text

Full details of mathematical models in joint-robust principal component analysis

SUPPLEMENTARY TABLES AND LEGENDS

Supplementary Table 1. (separate file)
Sample metadata. Table includes data taken during intake and over the course of the study.

Supplementary Table 2. (separate file)
ANCOM-BC differential abundance analysis results of cadaver skin metabolite log-ratio change over decomposition stages. Initial day 0 samples were used as the reference level and the intercept. Results include log-ratio changes of day 0 metabolites to early, active, and advanced decomposition stages, P-values, Holm–Bonferroni-corrected P-values (Q-values), standard errors, and W-values.

Supplementary Table 3. (separate file)
ANCOM-BC differential abundance analysis results of cadaver-associated soil metabolite log-ratio change over decomposition stages. Initial day 0 samples were used as the reference level and the intercept. Results include log-ratio changes of day 0 metabolites to early, active, and advanced decomposition stages, P-values, Holm–Bonferroni-corrected P-values (Q-values), standard errors, and W-values.

Supplementary Table 4. (separate file)
List of samples used to generate shotgun metagenomic data.

Supplementary Table 5.
Assembly statistics and GTDB taxonomic classification of genomic bins (metagenome-assembled genomes; MAGs) co-assembled from the metagenomic samples. Table includes completeness and contamination of each MAG.

Supplementary Table 6. (separate file)
TPM-normalized count abundance of MAGs within metagenomic samples.

Supplementary Table 7. (separate file)
Linear mixed-effects model statistics for testing response variable change of ATP per C-mol amino acids calculated from metagenomic data over ADD at each facility and a random intercept for each individual body to account for repeated measures to test whether the metabolism efficacy shifts within each facility. Formula: “ATPm ~ ADD + (1|body ID)”.

Supplementary Table 8. (separate file)
Linear mixed-effects model statistics for testing response variable change of ATP per C-mol carbohydrates calculated from metagenomic data over ADD at each facility and a random intercept for each individual body to account for repeated measures to test whether the metabolism efficacy shifts within each facility. Formula: “ATPm ~ ADD + (1|body ID)”.

Supplementary Table 9. (separate file)
Linear mixed-effects model statistics for testing response variable change of ATP per C-mol lipids calculated from metagenomic data over ADD at each facility and a random intercept for

each individual body to account for repeated measures to test whether the metabolism efficacy shifts within each facility. Formula: “ATPm ~ ADD + (1|body ID)”.

Supplementary Table 10.
Kruskal-Wallis rank sum test statistics for comparison of βNTI distance values between decomposition stages at each facility. The tests used were Kruskal-Wallis rank sum tests.

Test	Metric	Facility	Chi-squared	df	p-value
Kruskal Wallis	βNTI	FIRS	524.89	2	<2.2E-16
Kruskal Wallis	βNTI	STAFS	132.37	2	<2.2E-16
Kruskal Wallis	βNTI	ARF	123.48	2	<2.2E-16

Supplementary Table 11.
Dunn multiple comparison with Benjamini-Hochberg adjustment test statistics for comparison of βNTI distance values between decomposition stages at each facility.

Test	Metric	Facility	Comparison	Z	P.unadj	P.adj
Dunn	βNTI	FIRS	AC-AD to EA-AC	17.59	2.73E-69	4.1E-69
Dunn	βNTI	FIRS	AC-AD to PL-EA	20.80	3.97E-96	1.19E-95
Dunn	βNTI	FIRS	EA-AC to PL-EA	9.93	3.18E-23	3.18E-23
Dunn	βNTI	STAFS	AC-AD to EA-AC	7.65	1.99E-14	2.99E-14
Dunn	βNTI	STAFS	AC-AD to PL-EA	8.92	4.6E-19	1.38E-18
Dunn	βNTI	STAFS	EA-AC to PL-EA	6.85	7.41E-12	7.41E-12
Dunn	βNTI	ARF	AC-AD to EA-AC	2.78	5.58E-03	5.58E-03
Dunn	βNTI	ARF	AC-AD to PL-EA	10.98	4.62E-28	1.39E-27
Dunn	βNTI	ARF	EA-AC to PL-EA	9.64	5.54E-22	8.32E-22

Supplementary Table 12.
Kruskal-Wallis rank sum test statistics for comparison of metabolic resource overlap (MRO) and metabolic interaction potential (MIP) values between decomposition stages at each facility. The tests used were Kruskal-Wallis rank sum tests.

Test	Metric	Facility	Dataset	Chi-squared	df	p-value
Kruskal Wallis	MRO	FIRS	Co-occurrence	4.1188	1	0.04241
Kruskal Wallis	MIP	FIRS	Co-occurrence	61.795	1	3.811E-15
Kruskal Wallis	MRO	STAFS	Co-occurrence	181.24	2	<2.2E-16
Kruskal Wallis	MIP	STAFS	Co-occurrence	190.67	2	<2.2E-16
Kruskal Wallis	MRO	ARF	Co-occurrence	3.5887	2	0.1662
Kruskal Wallis	MIP	ARF	Co-occurrence	134.88	2	<2.2E-16
Kruskal Wallis	MRO	FIRS	Null/Random	0.3648	1	0.5459
Kruskal Wallis	MIP	FIRS	Null/Random	0.2267	1	0.634
Kruskal Wallis	MRO	STAFS	Null/Random	0.4032	2	0.8174
Kruskal Wallis	MIP	STAFS	Null/Random	4.3567	2	0.1132

Kruskal Wallis	MRO	ARF	Null/Random	3.2102	2	0.2009
Kruskal Wallis	MIP	ARF	Null/Random	1.0856	2	0.5811

Supplementary Table 13.
Dunn's multiple comparison with Benjamini-Hochberg adjustment test statistics for comparison of metabolic resource overlap (MRO) and metabolic interaction potential (MIP) values between decomposition stages at each facility.

Test	Metric	Facility	Comparison	Z	P.unadj	P.adj
Dunn	MRO	STAFS	Active-advanced	13.46	2.62E-41	7.85E-41
Dunn	MRO	STAFS	Active-early	6.63	3.42E-11	3.42E-11
Dunn	MRO	STAFS	Advanced-early	-6.84	8.2E-12	1.23E-11
Dunn	MIP	STAFS	Active-advanced	-10.24	1.36E-24	2.04E-24
Dunn	MIP	STAFS	Active-early	-13.14	1.84E-39	5.53E-39
Dunn	MIP	STAFS	Advanced-early	-2.91	3.65E-3	3.65E-3
Dunn	MRO	ARF	Active-advanced	-0.8	0.42	0.42
Dunn	MRO	ARF	Active-early	-1.89	0.06	0.18
Dunn	MRO	ARF	Advanced-early	-1.09	0.28	0.42
Dunn	MIP	ARF	Active-advanced	0.05	0.96	0.96
Dunn	MIP	ARF	Active-early	10.08	6.51E-24	1.95E-23
Dunn	MIP	ARF	Advanced-early	10.03	1.11E-23	1.67E-23

Supplementary Table 14. (separate file)
Number of predicted exchanges for cross-fed compounds at each facility during late decomposition. Late decomposition was defined as the advanced decomposition stage at STAFS and ARF and the active decomposition stage at FIRS.

Supplementary Table 15. (separate file)
Linear mixed-effects model statistics for testing response variable change of Generalized UniFrac PC1 distances calculated from 16S rRNA gene data over ADD at each facility with sampling site (i.e., soil adjacent to hip vs. soil control) as an independent variable (fixed effect) and a random intercept for each individual body to account for repeated measures. The models measure the sampling site and ADD variables individually and the interaction between the variables. The interaction between the variables was used to test whether the sampling sites respond differently to decomposition. Formula: "diversity metric ~ ADD * sampling site + (1|body ID)".

Supplementary Table 16. (separate file)
Linear mixed-effects model statistics for testing response variable change of ASV richness calculated from 16S rRNA gene data over ADD at each facility with sampling site (i.e., soil adjacent to hip vs. soil control) as an independent variable (fixed effect) and a random intercept for each individual body to account for repeated measures. The models measure the sampling site and ADD variables individually and the interaction between the variables. The interaction

between the variables was used to test whether the sampling sites respond differently to decomposition. Formula: “diversity metric ~ ADD * sampling site + (1|body ID)”.

Supplementary Table 17.
Permutational multivariate analysis of variance model statistics using vegan-R adonis in QIIME2 v. 2022.2 (permutations = 5000) with the combined multi-omics Joint-RPCA distance matrix as the response variable. Formula: “distance matrix ~ decomp_group * climate * facility * season”.

	Df	SumsOfSqs	MeanSqs	F.Model	R2	Pr(>F)
decomp_group	3	0.5099	0.1700	63.8083	0.0958	0.0002
climate	1	1.2560	1.2560	471.5303	0.2359	0.0002
facility	1	1.0828	1.0828	406.5141	0.2034	0.0002
season	3	0.8440	0.2813	105.6233	0.1585	0.0002
decomp_group:climate	3	0.0528	0.0176	6.6015	0.0099	0.0002
decomp_group:facility	3	0.0223	0.0074	2.7856	0.0042	0.0006
decomp_group:season	9	0.1156	0.0128	4.8220	0.0217	0.0002
climate:season	2	0.0668	0.0334	12.5446	0.0126	0.0002
facility:season	3	0.3794	0.1265	47.4766	0.0713	0.0002
decomp_group:climate:season	6	0.0084	0.0014	0.5282	0.0016	0.9756
decomp_group:facility:season	9	0.1065	0.0118	4.4426	0.0200	0.0002
Residuals	330	0.8790	0.0027	NA	0.1651	NA
Total	373	5.3234	NA	NA	1.0000	NA

Supplementary Table 18.
Permutational multivariate analysis of variance model statistics using vegan-R adonis in QIIME2 v. 2022.2 (permutations = 5000) with the 16S rRNA gene abundances RPCA distance matrix as the response variable. Formula: “distance matrix ~ decomp_group * climate * facility * season”.

	Df	SumsOfSqs	MeanSqs	F.Model	R2	Pr(>F)
decomp_group	3	0.2827	0.0942	36.8614	0.0873	0.0002
climate	1	0.6330	0.6330	247.5760	0.1954	0.0002
facility	1	0.5326	0.5326	208.3075	0.1644	0.0002
season	3	0.4822	0.1607	62.8620	0.1489	0.0002
decomp_group:climate	3	0.0329	0.0110	4.2953	0.0102	0.0002
decomp_group:facility	3	0.0717	0.0239	9.3497	0.0221	0.0002
decomp_group:season	9	0.0334	0.0037	1.4535	0.0103	0.0638
climate:season	2	0.0787	0.0394	15.3978	0.0243	0.0002
facility:season	3	0.1762	0.0587	22.9734	0.0544	0.0002
decomp_group:climate:season	6	0.0361	0.0060	2.3502	0.0111	0.0022
decomp_group:facility:season	9	0.0356	0.0040	1.5455	0.0110	0.0496
Residuals	330	0.8438	0.0026	NA	0.2605	NA
Total	373	3.2390	NA	NA	1.0000	NA

Supplementary Table 19.
Permutational multivariate analysis of variance model statistics using vegan-R adonis in QIIME2 v. 2022.2 (permutations = 5000) with the 18S rRNA gene abundances RPCA distance matrix as the response variable. Formula: “distance matrix ~ decomp_group * climate * facility * season”.

	Df	SumsOfSqs	MeanSqs	F.Model	R2	Pr(>F)
decomp_group	3	0.1078	0.0359	16.3211	0.0347	0.0002
climate	1	0.5048	0.5048	229.2283	0.1627	0.0002
facility	1	0.2779	0.2779	126.2109	0.0896	0.0002
season	3	0.8685	0.2895	131.4546	0.2799	0.0002
decomp_group:climate	3	0.0346	0.0115	5.2303	0.0111	0.0002
decomp_group:facility	3	0.0289	0.0096	4.3744	0.0093	0.0002
decomp_group:season	9	0.0913	0.0101	4.6081	0.0294	0.0002
climate:season	2	0.2433	0.1217	55.2429	0.0784	0.0002
facility:season	3	0.1768	0.0589	26.7664	0.0570	0.0002
decomp_group:climate:season	6	0.0172	0.0029	1.3019	0.0055	0.1882
decomp_group:facility:season	9	0.0250	0.0028	1.2638	0.0081	0.1822
Residuals	330	0.7267	0.0022	NA	0.2342	NA
Total	373	3.1029	NA	NA	1.0000	NA

Supplementary Table 20.
Permutational multivariate analysis of variance model statistics using vegan-R adonis in QIIME2 v. 2022.2 (permutations = 5000) with the MAG abundances RPCA distance matrix as the response variable. Formula: “distance matrix ~ decomp_group * climate * facility * season”.

	Df	SumsOfSqs	MeanSqs	F.Model	R2	Pr(>F)
decomp_group	3	0.3455	0.1152	72.3992	0.1023	0.0002
climate	1	0.9660	0.9660	607.3127	0.2860	0.0002
facility	1	1.0219	1.0219	642.4532	0.3025	0.0002
season	3	0.1352	0.0451	28.3228	0.0400	0.0002
decomp_group:climate	3	0.0200	0.0067	4.1916	0.0059	0.0002
decomp_group:facility	3	0.0111	0.0037	2.3341	0.0033	0.0128
decomp_group:season	9	0.0385	0.0043	2.6874	0.0114	0.0002
climate:season	2	0.0217	0.0109	6.8337	0.0064	0.0002
facility:season	3	0.2036	0.0679	42.6605	0.0603	0.0002
decomp_group:climate:season	6	0.0135	0.0022	1.4100	0.0040	0.1194
decomp_group:facility:season	9	0.0762	0.0085	5.3231	0.0226	0.0002
Residuals	330	0.5249	0.0016	NA	0.1554	NA
Total	373	3.3780	NA	NA	1.0000	NA

Supplementary Table 21.

Permutational multivariate analysis of variance model statistics using vegan-R adonis in QIIME2 v. 2022.2 (permutations = 5000) with the MAG gene abundances RPCA distance matrix as the response variable. Formula: "distance matrix ~ decomp_group * climate * facility * season".

	Df	SumsOfSqs	MeanSqs	F.Model	R2	Pr(>F)
decomp_group	3	0.8410	0.2803	34.4509	0.1090	0.0002
climate	1	1.6593	1.6593	203.9138	0.2151	0.0002
facility	1	1.0324	1.0324	126.8717	0.1338	0.0002
season	3	0.4349	0.1450	17.8133	0.0564	0.0002
decomp_group:climate	3	0.1043	0.0348	4.2718	0.0135	0.0008
decomp_group:facility	3	0.0154	0.0051	0.6310	0.0020	0.7473
decomp_group:season	9	0.1991	0.0221	2.7190	0.0258	0.0002
climate:season	2	0.0702	0.0351	4.3162	0.0091	0.0010
facility:season	3	0.5085	0.1695	20.8298	0.0659	0.0002
decomp_group:climate:season	6	0.0183	0.0031	0.3754	0.0024	0.9802
decomp_group:facility:season	9	0.1469	0.0163	2.0062	0.0190	0.0100
Residuals	330	2.6854	0.0081	NA	0.3480	NA
Total	373	7.7158	NA	NA	1.0000	NA

Supplementary Table 22.

Permutational multivariate analysis of variance model statistics using vegan-R adonis in QIIME2 v. 2022.2 (permutations = 5000) with the MAG gene module abundances RPCA distance matrix as the response variable. Formula: "distance matrix ~ decomp_group * climate * facility * season".

	Df	SumsOfSqs	MeanSqs	F.Model	R2	Pr(>F)
decomp_group	3	0.2805	0.0935	22.0621	0.0893	0.0002
climate	1	0.5196	0.5196	122.6151	0.1654	0.0002
facility	1	0.2022	0.2022	47.7039	0.0644	0.0002
season	3	0.1448	0.0483	11.3934	0.0461	0.0002
decomp_group:climate	3	0.0528	0.0176	4.1555	0.0168	0.0002
decomp_group:facility	3	0.0176	0.0059	1.3833	0.0056	0.1944
decomp_group:season	9	0.0664	0.0074	1.7405	0.0211	0.0138
climate:season	2	0.0680	0.0340	8.0263	0.0217	0.0002
facility:season	3	0.2676	0.0892	21.0527	0.0852	0.0002
decomp_group:climate:season	6	0.0304	0.0051	1.1942	0.0097	0.2603
decomp_group:facility:season	9	0.0927	0.0103	2.4307	0.0295	0.0006
Residuals	330	1.3984	0.0042	NA	0.4452	NA
Total	373	3.1411	NA	NA	1.0000	NA

Supplementary Table 23.
Permutational multivariate analysis of variance model statistics using vegan-R adonis in QIIME2 v. 2022.2 (permutations = 5000) with the soil metabolites abundance with predicted chemical abundances RPCA distance matrix as the response variable. Formula: “distance matrix ~ decomp_group * climate * facility * season”.

	Df	SumsOfSqs	MeanSqs	F.Model	R2	Pr(>F)
decomp_group	3	0.0278	0.0093	1.4870	0.0090	0.1442
climate	1	0.0591	0.0591	9.4983	0.0191	0.0004
facility	1	0.0740	0.0740	11.8883	0.0240	0.0002
season	3	0.4316	0.1439	23.1200	0.1397	0.0002
decomp_group:climate	3	0.0126	0.0042	0.6745	0.0041	0.7165
decomp_group:facility	3	0.0399	0.0133	2.1376	0.0129	0.0286
decomp_group:season	9	0.0581	0.0065	1.0371	0.0188	0.4095
climate:season	2	0.0725	0.0363	5.8288	0.0235	0.0002
facility:season	3	0.1279	0.0426	6.8544	0.0414	0.0002
decomp_group:climate:season	6	0.0164	0.0027	0.4383	0.0053	0.9644
decomp_group:facility:season	9	0.1153	0.0128	2.0593	0.0373	0.0058
Residuals	330	2.0533	0.0062	NA	0.6648	NA
Total	373	3.0885	NA	NA	1.0000	NA

Supplementary Table 24.
Test statistics for comparison of Joint-RPCA PC values from Axes 1 through 4 on metadata variables season, climate, decomposition stage, and facility. The tests used were two-tailed Kruskal-Wallis H tests and Mann-Whitney U tests and no multiple comparison adjustments.

Test	Axis	Factor	n_groups	test-statistic	test-statistic-value	p-value
Kruskal Wallis	1	season	4	H	218.48692	4.26E-47
Mann-Whitney U	1	climate	2	U	6429	0.00030241
Kruskal Wallis	1	facility	3	H	14.1469549	0.00084728
Kruskal Wallis	1	decomp_group	4	H	8.57783592	0.03546368
Kruskal Wallis	2	decomp_group	4	H	111.845888	4.40E-24
Kruskal Wallis	2	season	4	H	83.7979755	4.70E-18
Kruskal Wallis	2	facility	3	H	63.879984	1.34E-14
Mann-Whitney U	2	climate	2	U	11962	0.00021862
Kruskal Wallis	3	facility	3	H	280.164284	1.46E-61
Mann-Whitney U	3	climate	2	U	748	1.00E-28
Kruskal Wallis	3	decomp_group	4	H	13.2926913	0.00404456
Kruskal Wallis	3	season	4	H	6.05613856	0.10891068
Kruskal Wallis	4	facility	3	H	267.377664	8.70E-59
Mann-Whitney U	4	climate	2	U	18259	2.89E-33
Kruskal Wallis	4	season	4	H	12.1795546	0.00679272

Kruskal Wallis	4	decomp_group	4	H	9.09038997	0.02811293

Supplementary Table 25. (separate file)
Joint-RPCA PC2 correlations calculated between network feature nodes that correspond with late (i.e., active and advanced) decomposition soil.

Supplementary Table 26. (separate file)
Joint-RPCA PC2 correlations calculated between network feature nodes in initial, non-decomposition and early decomposition soil.

Supplementary Table 27. (separate file)
16S rRNA gene ASVs assigned to the same taxonomy as decomposer network taxa. Table includes the phylogenetic tree labels in Figure 4E, 150bp length ASVs and trimmed 100bp length ASVs used to explore ASV presence in other studies.

Supplementary Table 28. (separate file)
Presence of universal decomposers in possible human and terrestrial source environments in a few other studies. Table shows the average relative abundance of each decomposer ASV across each sample type. Average relative abundances were then summed for each decomposer genus.

Supplementary Table 29. (separate file)
Cross-feeding statistics for MAGs predicted as cross-feeders during late decomposition. Table includes GTDB taxonomic classification, number of reactions each MAG was considered the compound receiver and/or donor, and the percent responsible for all donations and acceptances during late decomposition. Late decomposition was defined as the advanced decomposition stage at STAFS and ARF and the active decomposition stage at FIRS.

Supplementary Table 30. (separate file)
Cross-feeding exchanges for *Oblitimonas alkaliphila* during late decomposition. *Oblitimonas alkaliphila* was not a predicted cross-feeder at FIRS during this timeframe. Table includes MAG ID and taxonomic classification of genomes involved in exchange, compounds exchanged, and computed interaction metrics.

Supplementary Table 31. (separate file)
Cross-feeding exchanges for L-arginine or ornithine during late decomposition. Table includes MAG ID and taxonomic classification of genomes involved in exchange, compounds exchanged, and computed interaction metrics.

Supplementary Table 32. (separate file)
Model validation results from predicting an independent test set of samples using the 16S rRNA gene at the SILVA database level-7 taxonomic rank random forest regression models for the skin of the hip and soil adjacent the hip. Errors are represented by MAE in ADD.

Supplementary Table 33. (separate file)

Presence of universal decomposers in a few other studies focused on mammalian decomposition environments. A search for the 35 universal PMI decomposer ASVs was conducted within each dataset. The relative abundance of each decomposer ASV was first averaged across all samples within a specific metadata category. The average relative abundances were then summed across each decomposer genus. Prevalence tables were constructed by summing the number of samples across a specific metadata category in which each universal decomposer ASV was present.

Supplementary Table 34. (separate file)
The average ADD per calendar day calculated for each cadaver at each facility. The average ADD per calendar day was calculated by dividing the final maximum ADD values by the total number of days (i.e., 21). The average ADD per day was calculated for each cadaver, season and facility, each climate type, and as a study-wide average.

Supplementary Table 35. (separate file)
The average ADD per calendar day calculated for each cadaver at each facility for the independent test set. The average ADD per calendar day was calculated by dividing the final maximum ADD values by the total number of sampling days. The average ADD per day was calculated for each cadaver, facility, and as a study-wide average.

Supplementary Table 36. (separate file)
Metabolite identification information for metabolites that had a predicted chemical formula or matched to a compound in the database library. When available, chemical formulas in the database library took precedence over predicted chemical formulas for calculating NOSC and major biochemical classes based on the molar H:C and O:C ratios.

Supplementary Table 37. (separate file)
Soil metabolite feature table normalized with sum normalization then scaled with pareto scaling. Table includes chemical formulas and major biochemical classes based on the molar H:C and O:C ratios.

Supplementary Table 38. (separate file)
Skin metabolite feature table normalized with sum normalization then scaled with pareto scaling. Table includes chemical formulas and major biochemical classes based on the molar H:C and O:C ratios.

Supplementary Table 39. (separate file)
Sample metadata for machine learning independent test set. Table includes data taken during intake and over the course of the study.

SUPPLEMENTARY TEXT

Methods

Preprocessing. Prior to joint factorization, we first split the data into training j_{train} and testing j_{test} samples set from the total set of shared samples k across all N input data matrix $X_{ij}^{1}, X_{ij}^{2}, \ldots, X_{ij}^{N}$. Each matrix X_{ij}^{k} is then transformed, through the robust centered log-ratio transformation (robust-clr) to center the data around zero and approximate a normal distribution [1].

$$rclr\left(x\right) = \left[\log\frac{x_1}{g_r\left(x\right)}, ..., \log\frac{x_D}{g_r\left(x\right)}\right] \tag{1}$$

$$g_r\left(x\right) = \left(\prod_{i\in\Omega_x} x_i\right)^{1/|\Omega_x|} \tag{2}$$

where x_i is the abundance of feature (e.g., microbe, metabolite, or gene) i, Ω_x is the set of observed microbes in sample x and $g_r\left(x\right)$ is the geometric mean only defined on microbes with abundance > 0. Unlike the traditional clr transformation, the robust-clr handles the sparsity often found in biological data without requiring imputation. The rclr transformation is applied to the training and test set matrices $rclr(X_{ij_{train}}^{k})$ and $rclr(X_{ij_{test}}^{k})$ independently to give $Y_{ij_{train}}^{k}$ and $Y_{ij_{test}}^{k}$.

Joint matrix factorization. The joint factorization used here is built upon the OptSpace matrix completion algorithm which is a singular value decomposition (SVD) optimized on a local manifold [2, 1].

$$\min_{\boldsymbol{U_{shared}}, \boldsymbol{V_k}} \frac{\sum_{k=1}^{N}\left|\Lambda\left(\boldsymbol{Y^k} - \boldsymbol{U_{shared}}\boldsymbol{S}\boldsymbol{V^{k^T}}\right)\right|_2^2}{N} \tag{3}$$

where $\boldsymbol{U_{shared}}$ is the matrix being estimated across the shared samples of all input matrices, $\boldsymbol{V^k}$ are the matrices being estimated corresponding to each respective to input matrix, and $\boldsymbol{S}$ is analogous to a matrix of shared eigenvalues across all input matrices. For each matrix $\boldsymbol{Y^k}$ the observed values and Λ is a function such that the errors between $\boldsymbol{Y^k}$ and $\boldsymbol{U_{shared}}\boldsymbol{S}\boldsymbol{V^{k^T}}$ are only computed on the nonzero entries and then averaged for each matrix, such that the minimized shared estimated matrices $\boldsymbol{U_{shared}}$ and $\boldsymbol{S}$ are optimized across all matrices. The minimization is performed across iterations by gradient decent. To ensure the rotation of the estimated matrices are consistent, $\boldsymbol{U_{shared}}$ and $\boldsymbol{S}$ are recalculated at each iteration by

$$\boldsymbol{U_{shared}}\boldsymbol{S}\boldsymbol{U_{shared}} = SVD\left(\frac{\sum_{k=1}^{N}\boldsymbol{U^k}\boldsymbol{U^{k^T}}}{N}\right) \tag{4}$$

where $\boldsymbol{U^k}$ is the update for each estimated matrix during that iteration. For each $\boldsymbol{V^k}$ iteration update we define $\boldsymbol{W^k}$ given by

$$\boldsymbol{W^k} = (\boldsymbol{S}\boldsymbol{V^{k^T}})^T \tag{5}$$

In order to prevent over fitting of the joint-factorization cross validation of the reconstruction can be performed. In this case, all of the previously described minimization is performed on only the training set data. The test set data is then projected into the same space using the training set data estimated matrices and the reconstruction of the test data is calculated, given by:

$$cross\text{-}validation\ error = \frac{\sum_{k=1}^{N} \left| \Lambda \left(\boldsymbol{Y^k_{test}} - (\boldsymbol{Y^k_{test}} \boldsymbol{V^k_{train}}^{\boldsymbol{T}}) \boldsymbol{W_{train}} \right) \right|_2^2}{N} \tag{6}$$

Through this it can be ensured that the minimization error of the training data estimations also minimizes that of the test set data, which is not incorporated into those estimates on each iteration. After the training data estimates are finalized the test set samples can again be projected into the final output to prevent those samples from being lost. The covariance of all features across all input matrices is calculated from the final estimated matrices by

$$feature\ covariance\ matrix = \begin{bmatrix} \boldsymbol{W_1} \\ \boldsymbol{W_2} \\ \dots \\ \boldsymbol{W_N} \end{bmatrix} \begin{bmatrix} \boldsymbol{W_1} \\ \boldsymbol{W_2} \\ \dots \\ \boldsymbol{W_N} \end{bmatrix}^T \tag{7}$$

Finally, here we treat the Joint-RPCA with only one input matrix X^1_{ij} as the original RPCA [1] but with the additional benefit of the addition of cross-validation for comparison across other methods.

References

[1] C. Martino, J. T. Morton, C. A. Marotz, L. R. Thompson, A. Tripathi, R. Knight, K. Zengler, A novel sparse compositional technique reveals microbial perturbations, mSystems 4 (2019).

[2] R. H. Keshavan, S. Oh, A. Montanari, Matrix completion from a few entries, in: 2009 IEEE International Symposium on Information Theory.
