# Supplementary material for: A conserved interdomain microbial network underpins cadaver decomposition despite environmental variables
Source: Nat Microbiol. 2024 Feb 12;9(3):595–613. doi: 10.1038/s41564-023-01580-y (PMC10914610; doi:10.1038/s41564-023-01580-y)
Supplement: Supplementary file 2 — Reporting Summary [file 41564_2023_1580_MOESM2_ESM.pdf]

nature portfolio

Corresponding author(s): Metcalf

Last updated by author(s): Dec 5, 2023

Reporting Summary

Nature Portfolio wishes to improve the reproducibility of the work that we publish. This form provides structure for consistency and transparency in reporting. For further information on Nature Portfolio policies, see our Editorial Policies and the Editorial Policy Checklist.

Statistics

For all statistical analyses, confirm that the following items are present in the figure legend, table legend, main text, or Methods section.

n/a	Confirmed	
☐	☒	The exact sample size (*n*) for each experimental group/condition, given as a discrete number and unit of measurement
☐	☒	A statement on whether measurements were taken from distinct samples or whether the same sample was measured repeatedly
☐	☒	The statistical test(s) used AND whether they are one- or two-sided<br>*Only common tests should be described solely by name; describe more complex techniques in the Methods section.*
☐	☒	A description of all covariates tested
☐	☒	A description of any assumptions or corrections, such as tests of normality and adjustment for multiple comparisons
☐	☒	A full description of the statistical parameters including central tendency (e.g. means) or other basic estimates (e.g. regression coefficient) AND variation (e.g. standard deviation) or associated estimates of uncertainty (e.g. confidence intervals)
☐	☒	For null hypothesis testing, the test statistic (e.g. *F*, *t*, *r*) with confidence intervals, effect sizes, degrees of freedom and *P* value noted<br>*Give P values as exact values whenever suitable.*
☒	☐	For Bayesian analysis, information on the choice of priors and Markov chain Monte Carlo settings
☒	☐	For hierarchical and complex designs, identification of the appropriate level for tests and full reporting of outcomes
☒	☐	Estimates of effect sizes (e.g. Cohen's *d*, Pearson's *r*), indicating how they were calculated

Our web collection on statistics for biologists contains articles on many of the points above.

Software and code

Policy information about availability of computer code

Data collection	No software was used for data collection.
Data analysis	QIIME2 analysis platform versions 2020.2, 2020.8, 2022.2, and 2023.5. Deblur denoising method QIIME2 version 2020.8.0 and standalone version (v1.1.1). RESCRIPt (v2022.8.0). Emperor (v2022.8.0). cutadapt (v4.4). Jupyter Lab (v4.0.5). Python (v3.8.16), MEGAHIT (v1.2.9). MetaBAT2 (v2.12.1). checkM (v1.1.2). MMseq2 (v13). dRep (v2.6.2). GTDB-tk (v2.0.0, r207). DRAM (v1.0.0). bowtie2 (v2.3.5). samtools (v1.9). BBMap (v.38.90). CoverM (v0.3.2). HiOrCo (v1.0.0). SMETANA (v1.0.0). CarveMe (v1.5.1). COBRA toolbox (v3.0). Feature-Based Molecular Networking (FBMN) workflow version 28.2. MZMINE2 (v2.53). SIRIUS+ZODIAC (v4.4). calour (v2018.5.1). scikit-learn (v0.24.2). ANCOM-BC (QIIME2 v.2022.2 version). Redbiom (v0.3.9). EMPress (v1.2.0). Analysis code, intermediate files, and metadata are publicly available on Github (https://github.com/Metcalf-Lab/2023-Universal-microbial-network). The complete mathematical algorithms for Joint-Robust Principal Component Analysis (Joint-RPCA) can be found in Supplementary Text.

For manuscripts utilizing custom algorithms or software that are central to the research but not yet described in published literature, software must be made available to editors and reviewers. We strongly encourage code deposition in a community repository (e.g. GitHub). See the Nature Portfolio guidelines for submitting code & software for further information.

Data

Policy information about availability of data

All manuscripts must include a data availability statement. This statement should provide the following information, where applicable:

- Accession codes, unique identifiers, or web links for publicly available datasets
- A description of any restrictions on data availability
- For clinical datasets or third party data, please ensure that the statement adheres to our policy

Raw amplicon and metagenomic sequencing data, and sample metadata are available on the QIITA open-source microbiome study management platform under study 14989 and ENA accession PRJEB62460 (ERP147550). Dereplicated MAGs and DRAM output can be found publicly on Zenodo (DOI:10.5281/zenodo.7843104; https://zenodo.org/record/7938240) and NCBI BioProject PRJNA973116. The mass spectrometry data were deposited on the MassIVE public repository (accession numbers: MSV000084322 for skin samples and MSV000084463 for soil samples). The molecular networking job can be publicly accessed at https://gnps.ucsd.edu/ProteoSAFe/status.jsp?task=1c73926f2eb5409985cc2e136062db2f. The GNPS database was accessed through https://gnps.ucsd.edu/. The GreenGenes2 database can be found at https://ftp.microbio.me/greengenes_release/. SILVA databases can be found at https://www.arb-silva.de/documentation/release-1381/. The Earth Microbiome Project data and American Gut Project data can be found on EBI under accessions ERP125879 and ERP012803, respectively. 16S rRNA gene amplicon sequence data files from Metcalf et al. 20166, Carter et al. 201525, Weiss et al. 201524, Thompson et al. 201769, McDonald et al. 201890, Deel et al. 202264, and Kodama et al. 201991, were obtained from QIITA92 under study IDs 10141 - 10143, 1609, 13114, 10317, 13301, and 11204, respectively. Data from Dangerfield et al. 202016 was obtained from the NCBI Sequence Read Archive under BioProject PRJNA525153. Data from the Shukla et al. 201826 study was obtained from the Max Planck Society Edmond repository (https://edmond.mpdl.mpg.de/dataset.xhtml?persistentId=doi:10.17617/3.UV4FBN). The GTDB data can be accessed at https://data.gtdb.ecogenomic.org/releases/.

Research involving human participants, their data, or biological material

Policy information about studies with human participants or human data. See also policy information about sex, gender (identity/presentation), and sexual orientation and race, ethnicity and racism.

Reporting on sex and gender	Sex is reported for each donor within the metadata (Table S1). Gender (identity/presentation) was not collected.
Reporting on race, ethnicity, or other socially relevant groupings	No socially constructed or socially relevant categorization variables were collected.
Population characteristics	Cause of death if known, initial condition, weight before placement, age in years if known, estimated age if not known, sex, donor storage type, days donor was stored, time since death to cooling, and placement head direction can be found within the metadata (Table S1). Population estimated age was minimum: 56, maximum: 97, mean: 71.2, and median: 69.
Recruitment	Donors are recruited through willed-body donation to the forensic anthropology facilities. Inclusion criteria for the remains were specified prior to experiment start and required that the remains were in the fresh stage of decomposition and had not been frozen (and not extensively cooled) or autopsied prior to placement at the facility.
Ethics oversight	The Institutional Review Board, the Protection of Human Subjects Committee, either determined review was not required or granted exempt status for donors at each respective facility since the proposed research does not involve human subjects as defined by federal regulations.

Note that full information on the approval of the study protocol must also be provided in the manuscript.

Field-specific reporting

Please select the one below that is the best fit for your research. If you are not sure, read the appropriate sections before making your selection.

☐ Life sciences ☐ Behavioural & social sciences ☒ Ecological, evolutionary & environmental sciences

For a reference copy of the document with all sections, see nature.com/documents/nr-reporting-summary-flat.pdf

Ecological, evolutionary & environmental sciences study design

All studies must disclose on these points even when the disclosure is negative.

Study description	Outdoor experiments on 36 human cadavers were conducted at three willed-body donation facilities: FIRS, STAFS, and ARF. Three deceased human subjects were placed, supine and unclothed, on the soil surface in the spring, summer, fall, and winter over the years of 2016 and 2017 at each facility (N = 36). Longitudinal, daily sampling was performed on each donor for 21 days.
Research sample	Skin and soil microbiome samples.
Sampling strategy	No pre-calculated sample size was used. Sampling strategy was based on previous research (Metcalf et al. 2016) and chosen to obtain longitudinal data in triplicate within each season and facility.

Data collection	We sampled the skin surface of the head and torso near the hip along with gravesoils (soils associated with decomposition) associated with each skin site over 21 days of decomposition. Control soil samples were taken of the same soil series and horizon that are not associated with body decomposition (known past or present) from areas within or just outside each facility. We collected swabs of 756 non-decomposition soil (controls), 756 gravesoil near the hip and 756 gravesoil near the face, 756 hip skin, and 756 face skin samples (N = 3,780). Data was collected by staff and students at each respective facility.
Timing and spatial scale	Three deceased human subjects were placed, supine and unclothed, on the soil surface in the spring, summer, fall, and winter over the years of 2016 and 2017 at each facility (N = 36). Sampling on each donor was performed daily for 21 days. Placement dates can be seen on Figures S1 and S2.
Data exclusions	Data were only excluded if they failed extraction, sequencing, or quality control.
Reproducibility	All raw data and analysis pipelines are publicly available.
Randomization	Donors were used if they fit the criteria for selection. Donors are used by a "first-come-first-serve" bases to obtain random sampling of the donor population.
Blinding	Samples needed to be unblinded so that during analysis the appropriate samples could be matched to the metadata and so that repeated measures could be grouped together.

Did the study involve field work? ☒ Yes ☐ No

Field work, collection and transport

Field conditions	Before placement, STAFS performs minimal removal of vegetation including raking of leaves and removal of shrubbery, and bodies placed at STAFS were placed in cages made of 1 cm x 1 cm wire fences and wooden frames to prevent vertebrate scavenging. The ARF and FIRS do not remove vegetation or place bodies under cages as standard protocol. Furthermore, bodies were placed no closer than 2.5 meters between sternum midpoints. Average daily temperatures were collected from the National Centers for Environmental Information (NCEI) website (https://www.ncei.noaa.gov/) and monthly total precipitation accumulation over the course of the study was collected from the Weather Underground website (https://www.wunderground.com/) from local weather stations: Grand Junction Regional Airport Station, McGhee Tyson Airport Station, and Easterwood Airport Station. Data can be viewed in Supp. Figures S1 and S2.
Location	Forensic Investigation Research Station (FIRS), Grand Junction, CO, USA (lat: 39.08, long: -108.55). Anthropology Research Facility (ARF), Knoxville, TN, USA (lat: 35.940959, long: -83.939774). Southeast Texas Applied Forensic Science Facility (STAFS), Huntsville, TX, USA (lat: 30.745, long: -95.476).
Access & import/export	Anthropological research facilities FIRS, ARF, and STAFS are owned and operated by Colorado Mesa University, University of Tennessee, and Same Houston State University, respectively.
Disturbance	Soil was sampled via cotton swabs and collecting small amounts in 50mL conical tubes to minimize disturbance.

Reporting for specific materials, systems and methods

We require information from authors about some types of materials, experimental systems and methods used in many studies. Here, indicate whether each material, system or method listed is relevant to your study. If you are not sure if a list item applies to your research, read the appropriate section before selecting a response.

Materials & experimental systems

n/a	Involved in the study	
☒	☐	Antibodies
☒	☐	Eukaryotic cell lines
☒	☐	Palaeontology and archaeology
☒	☐	Animals and other organisms
☒	☐	Clinical data
☒	☐	Dual use research of concern
☒	☐	Plants

Methods

n/a	Involved in the study	
☒	☐	ChIP-seq
☒	☐	Flow cytometry
☒	☐	MRI-based neuroimaging
